# Impact of Cationic and Neutral Clay Minerals’ Incorporation in Chitosan and Chitosan/PVA Microsphere Properties

**DOI:** 10.1021/acsami.4c22323

**Published:** 2025-03-28

**Authors:** Suelen Delfino Souza, Hugo Fernando Meira dos Santos, Larissa Fernandes Bonfim, Iara Silva Squarisi, Tábata Esperandim, Liziane Marçal, Denise Crispim Tavares, Emerson Henrique de Faria

**Affiliations:** Grupo de Pesquisas em Materiais Lamelares Híbridos (GPMatLam), Universidade de Franca (Unifran), Av. Dr. Armando Salles Oliveira, 201 Parque Universitário, Franca, SP 14404-600, Brazil

**Keywords:** biopolymers, kaolinite, saponite, adsorption, heavy metal, sensing

## Abstract

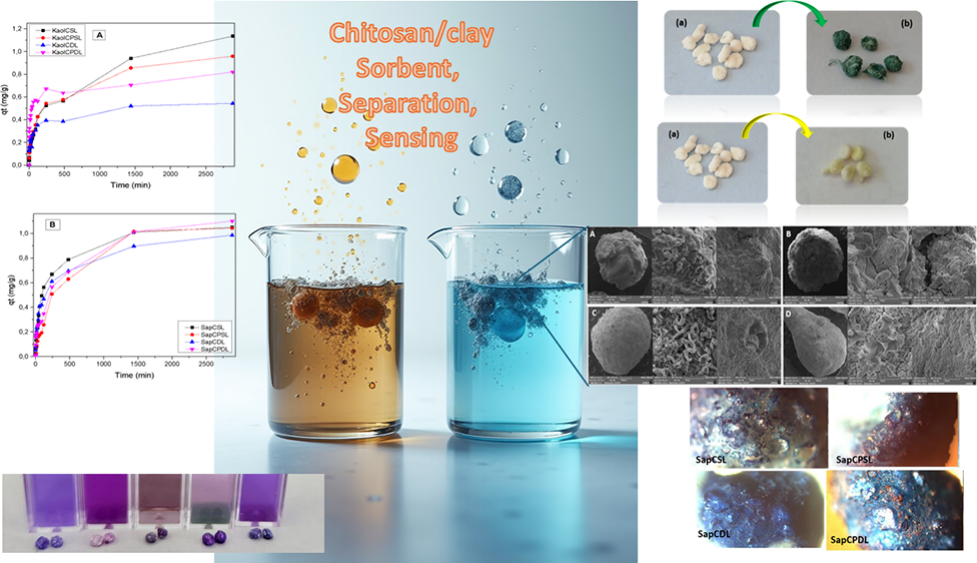

This study investigates the synthesis of chitosan and chitosan/poly(vinyl alcohol) (PVA) microspheres incorporated with kaolinite and synthetic saponite clays. The microspheres were prepared using a two-step process: (i) reticulation of chitosan and chitosan/PVA with kaolinite or saponite to form a clay-biopolymer matrix and (ii) further reticulation of chitosan/PVA to produce double-layered microspheres. The resulting materials were characterized using FTIR, XRD, thermal analysis and SEM. Their properties were evaluated for water uptake, cation exchange capacity, specific surface area, acid stability, and methylene blue, Cr^3+^, Cr^6+^, and Ni^2+^ adsorption. XRD analysis confirmed a successful polymer interaction with both clay structures. Cationic saponite clay favored clay dispersion, resulting in more homogeneous microspheres. Swelling tests revealed that chitosan-kaolinite microspheres exhibited 75% swelling, while chitosan-PVA-kaolinite microspheres showed 70% swelling, attributed to structural changes induced by PVA. Adsorption tests demonstrated that *KaolCSL* microspheres removed 53% of methylene blue (MB) and 82% of Ni^2+^, while *SapCPSL* microspheres exhibited superior removal of Cr^3+^ (91%), Cr^6+^ (19%), and silver nanoparticles (>90%). Biocompatibility assessments using zebrafish and HaCat cells showed no mortality or genotoxicity, with a 38% increase in cell viability for Cr-loaded microspheres. These results suggest that the use of modified clay-biopolymer microspheres can be an effective, low-cost solution for water purification and wastewater treatment.

## 1 Introduction

Biodegradable clay/biopolymers are commonly employed in controlled release systems and, as such, are extensively used in pharmaceuticals, adsorbents, and crop treatments.^1^ Chitosan (C) and poly(vinyl alcohol) (PVA) biopolymers, isolated and mixed as copolymers, are an attractive alternative to other biomaterials because of their significant physicochemical and biological activities. Clay/biopolymer microspheres are promising as carrier systems for drugs and vaccines (via oral, mucosal, and transdermal routes) and also for agricultural products such as fertilizers and pesticides. In this sense, controlling the swelling capacity of the hydrogels and improving the extremely fragile nature of the microspheres under different environmental conditions, such as temperature, acidity, and basicity, are the key challenges that need to be overcome to allow the widespread use of clay/biopolymer microspheres for controlled or sustained release.

The synthesis of hybrid materials allows changes in various surface characteristics of clay minerals (e.g., surface loading, roughness, reactivity, and surface energy). In this sense, methods such as the formation of microspheres with inorganic and biopolymeric compounds have been investigated for the development of new properties of clay minerals. The synthesis of materials with new or improved properties based on individual components (biopolymers and clay minerals) is important. Therefore, the addition of layered silicates such as natural (kaolinite) and synthetic clay minerals (saponite) is a promising alternative to increase the contaminant and water sorption capacity of these materials.^2^

Different polymers and copolymers have been identified for their important contribution to the adsorption of contaminants, as well as targeted, site-specific, and smart drug delivery systems. The goal of recent research is to synthesize and coprocess natural polymers with synthetic polymers, resulting in copolymers that have the advantages of both polymers, and sometimes unique properties.^3^

Chitosan is a natural biopolymer that is biodegradable, biocompatible, and nontoxic. Chitosan is obtained by a deacetylation reaction from chitin, which is found in the exoskeletons of crustaceans. Chitosan has interesting physicochemical properties, such as the presence of reactive hydroxyl and amino groups and a high positive charge under acidic conditions. Poly(vinyl alcohol) is a water-soluble synthetic polymer that has extremely good chemical and physical properties, such as film-forming ability.^2^ Several applications have been reported for copolymers including hydrogel membranes, burn dressings, controlled drug delivery, and tissue engineering. Natural polymers have good biocompatibility and biodegradability, but poor mechanical properties. This drawback can be overcome by using synthetic routes to modify natural biopolymers, such as by combining synthetic and natural biopolymers to obtain modified polymers with tunable properties to form microspheres. Their potential as drug carriers can be investigated by analyzing their drug release behavior under different conditions. The aim of this work is to contribute to the development of efficient and sustainable systems for adsorption, drug delivery, and other applications, using biodegradable and environmentally friendly materials.

Isolated and combined copolymers have been shown to exhibit poor mechanical and thermal stabilities. To address this issue, organic matrices can be combined with inorganic layered silicates, such as kaolinite and saponite. These combinations, depending on the chosen synthetic route, can result in conventional composites or nanocomposites (intercalated or exfoliated) with unique properties.

Kaolinite, with the theoretical formula Al_2_Si_2_O_5_(OH)_4_ and basal interlayer spacing of 7.1 Å, is a 1:1 or TO type clay mineral. It is formed by combining sheets of SiO_4_ tetrahedra (T) and Al(OH)_6_ octahedra (O) in a 1:1 proportion. The lamellae remain attached to each other because they share common oxygen atoms, giving rise to the structure of the clay mineral.^4^

Saponite is a smectite clay, a phyllosilicate, or layered TOT silicate that has a layered lattice structure in which two-dimensional oxoanions are separated by layers of hydrated cations. The oxygen atoms define upper and lower sheets enclosing tetrahedral sites, where a central sheet in the saponite structure is composed of brucite (Mg(OH)_2_) and gibbsite (Al(OH), enclosing octahedral sites. The minimal theoretical formula for saponite is M_*x*/*n*_^*n*+^ [Mg_6_][Si_8_ – *x*Al_*x*_]O_20_(OH)_4_*n*H_2_O (with *x* = 0.4 to 1.2; M_*x*/*n*_^*n*+^ = counterion).^5^

Here we describe an investigation of the effect of neutral or cationic synthetic clay minerals on the synthesis of C-clay and C-PVA-clay microspheres composed of single or double layers of both polymers. In addition, we evaluated various microspheres obtained.

The combination of clay minerals, chitosan, and polymers can result in hybrid materials with optimized properties for pollutant adsorption. The interplay of the surface of the clays, the functional groups of chitosan, and the specific properties of the polymers can lead to highly efficient and selective systems for the removal of potentially toxic trace metals, nanoparticles, and dyes from aquatic or industrial environments while also enabling the development of sustainable materials for the treatment of contaminated water.

## 2 Experimental Section

### 2.1 Materials

Kaolin came from the municipality of São Simão in the state of São Paulo, Brazil, and was supplied by the mining company Darcy R. O. Silva & Cia. Kaolinite, with the ideal chemical formula Al_2_Si_2_O_5_(OH)_4_, was used as the natural neutral clay source, where hydroxyl groups contribute to its unique surface properties and interactions. It is classified as the ball-clay type, characterized by fine granulometry and richness of hexagonal kaolinite. It was previously characterized by us, resulting in deduction of the following chemical formula: Si_2.0_Al_1.96_Fe_0.03_Mg_0.01_K_0.02_Ti_0.03_O_7.06_.^4^

The materials used were: chitosan from shrimp shells (C_12_H_24_N_2_O_9_) (CAS 9012-76-4) (Sigma-Aldrich), practical grade, with molecular weight compatible with chitosan having medium molecular weight, fragments estimated by MALDI-TOF (523, 525, 527, 550, 599, 637, 675, 699, 713, 760, 789, 827, 851, 853, 965, and 1004 *m*/*z*) (Figure S5). The values found are comparable with previous results from literature data.^6^ Additionally, the average molecular weight (Mw) was 280,000 Da, determined by gel permeation chromatography, with deacetylation degree >75%; poly(vinyl alcohol) (PVA) (C_2_H_4_O)x, molecular weight: 89,000 to 98,000 Da (CAS 9002-89-5) (Sigma-Aldrich, 98%); acetic acid (CH_3_COOH) (CAS 64-19-7) (Synth, 99.9%); sodium hydroxide (NaOH ≥ 98%) (CAS 1310-73-2) (Sigma-Aldrich, 100%); sodium silicate solution (Na_2_O(SiO_2_)_*x*_*x*H_2_O 27% w/v, Sigma-Aldrich) (CAS 338443); sodium bicarbonate (NaHCO_3_, 99.7%, Sigma-Aldrich) (CAS 144-55-8); aluminum chloride hexahydrate (AlCl_3_·6H_2_O, 99%) (CAS 7784-13-6); magnesium chloride hexahydrate (MgCl_2_·6H_2_O, 99%) (CAS 7791-18-6, Sigma-Aldrich); methylene blue hydrate (C_16_H_18_ClN_3_S *x*H_2_O) (CAS: 61-73-4) (Sigma-Aldrich, 97%); nickel(II) chloride hexahydrate (NiCl_2_·6H_2_O) (CAS 7791-20-0) (Sigma-Aldrich, 99%); chromium trichloride hexahydrate (CrCl_3_ 6H_2_O) (CAS 10060-12-5) (Sigma-Aldrich, 98%); potassium dichromate (K_2_Cr_2_O7) (CAS 7778-50-9) (Perfyl Tech, 99%); **c**obalt chloride hexahydrate (CoCl_2_ 6H_2_O) (CAS 7791-13-1) (Cinética, 98%); and copper(II) chloride (CuCl_2_) (CAS 7447-39-4) (Dinâmica, 97%).

### 2.2 Synthesis of Synthetic Saponite

Samples were prepared according to the method adapted from Trujillano et al. using the microwave-assisted synthesis method to optimize the time interval to synthesize saponite, and also adapting the magnetic stirring in each Teflon flask.^7^ A solution containing NaOH (0.09 mol) and NaHCO_3_ (0.08 mol) was prepared, which provided a slightly alkaline medium. A solution of cations (solution A) was prepared by adding 0.035 mol of sodium silicate solution (25 °C), which had a pH near 13. The resulting mixture was submitted to vigorous magnetic stirring. In another container, solution B was prepared by dissolving stoichiometric amounts of aluminum chloride hexahydrate (0.005 mol) and magnesium chloride hexahydrate (0.030 mol) in 50.0 mL of deionized water. The Si/Al and Si/Mg molar ratios in the reaction medium were 7:1 and 7:6, respectively. Solution B was slowly added to solution A under vigorous magnetic stirring, resulting in the formation of a white gel. The system was then heated to 180 °C for 30 min in an Ethos Easy microwave digestion platform using individual magnetic stirring, a system adapted exclusively for inorganic synthesis, with potency of 1500 W, heating rate of 10 °C/min until 180 °C, maintained during 30 min to guarantee the clay crystallization. The comparison between conventional hydrothermal and fast microwave-assisted synthesis was previously studied by Trujillano et al.^7^ and improved by us in terms of reduction of time required for crystallization (from 2 h to 30 min). The data are presented in the Supporting Information, Figures S1 and S2A,B.

### 2.3 Preparation and Characterization of Clay/Biopolymer Microspheres

#### 2.3.1 Preparation of Clay/Biopolymers with a Single Layer

To synthesize the microspheres, chitosan-clay (kaolinite or synthetic saponite) and C-PVA/clay with a single or double layer, 25 mL of PVA solution (2% w/v) was added to 25 mL of 2% C (w/v) solution, which was previously suspended in 100 mL of 3% acetic acid (w/v) and vigorously stirred mechanically. The resulting solution was constantly heated to 60 °C for 2 h. After this process, 7 g of kaolinite or saponite was added to the solution and mechanically stirred for 24 h. The microspheres with a single layer were produced by dropwise phase inversion of the gel obtained previously in an 8% (w/v) sodium hydroxide solution. The microspheres were then washed several times with deionized water until the supernatant’s pH was near 7 (Figure S3). The same basic NaOH solution was recovered and employed to synthesize all microspheres, as described below.

C-PVA/clay microspheres were prepared with either a single coating layer (clay incorporated in a single chitosan-PVA layer) or a double coating layer (an additional outer chitosan-PVA layer applied to the clay-loaded microspheres).

#### 2.3.2 Preparation of Clay/Biopolymers with a Double Layer

To prepare double-layered biopolymer/clay microspheres, the gel obtained as described in Section 2.3.1 was dried in an oven at 60 °C for 24 h and then ground into a powder using a mortar and pestle. The resulting powder was added to gels of C or C-PVA at the same concentrations as previously described. Finally, the gels were added dropwise to a sodium hydroxide solution. The microspheres were washed multiple times with deionized water until the supernatant’s pH was close to 7. The microspheres derived from kaolinite or saponite containing chitosan and PVA are designated as KaolC or SapC and KaolCP or SapCP, respectively, with the terms sl and dl used to indicate single or double layers (Figure S4).

This work is aligned with Sustainable Development Goal (SDG) 9 by fostering innovation and promoting sustainable industrial processes through the development of microwave-assisted synthesis and low-carbon technologies. Additionally, the recovery and reuse of reagents contribute to SDG 12 (Responsible Consumption and Production) by minimizing waste and enhancing resource efficiency. The use of water as a solvent and energy-efficient synthesis also support SDG 13 (Climate Action) by reducing the environmental footprint of the process. These efforts demonstrate a commitment to advancing sustainable scientific practices in line with the global sustainability goals.

### 2.4 Water Uptake (Swelling)

The water uptake capacity of the microspheres was determined by immersing them in a pH 7 water solution for 24 h. Specifically, 0.05 g of each microsphere was placed in 5.0 mL of water (10 g L^–1^ concentration). The initial weight of the microspheres was recorded, and the final weight was determined after blotting with a paper towel using eq 1:
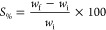
1where *S*_%_ = microsphere swelling degree; *w*_f_ = final microsphere weight (after swelling); *w*_i_ = initial microsphere weight.

### 2.5 Acid Stability (Kinetic Evaluation)

The stability of the microspheres containing clay/biopolymer composites in 0.1 M of HCl solution was evaluated by incubating 0.5% w/v suspensions of the microspheres in 0.1 M of HCl for 24 h, with measurements taken at regular intervals (0–250 min) to determine the swelling degree. The transmittance of the solution that was in contact with the microspheres was measured at λ = 500 nm using a Hewlett-Packard UV–vis spectrophotometer model 8453, as previously described by Qi et al.^8^ Additionally, a complementary study was conducted to monitor the mass loss of C, PVA, or clay in the medium, also using eq 1.

### 2.6 Adsorption Studies of Ni^2+^, Cr^3+^, Cr_2_O_7_^–^, Methylene Blue, and AgNP

A total of 10 g L^–1^ of microspheres was added to a 100 mL Erlenmeyer flask containing 50 mL of each solution with initial concentrations of 10 mg L^–1^ for MB, 100 mg L^–1^ for Ni^2+^, 1000 mg L^–1^ for Cr^3+^, 60 mg L^–1^ for Cr^6+^ and 1.68 mg L^–1^ for AgNP, to study the kinetic profile. The experiments were performed at 30 °C for 48 h. Samples were collected at predetermined intervals and analyzed by measuring the absorbance at λ = 664 nm for MB, λ = 584 nm for Ni^2+^, λ = 430 nm (Figure S6) for Cr^3+^, λ = 350 nm for Cr^6+^, and λ = 410 nm for AgNP using a Hewlett-Packard UV–vis spectrophotometer model 8453. The adsorption capacity and removal efficiency at the predetermined times were calculated using eqs 2 and 3 at time *t*.
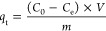
2
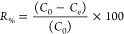
3where *q*_t_ = adsorption capacity (mg g^–1^); *C*_0_ = initial concentration of contaminant (mg L^–1^); *C*_e_ = equilibrium concentration of contaminant (mg L^–1^); and *R*_%_ = removal efficiency.

The concentrations chosen for this study represent typical contamination levels found in industrial and environmental wastewater, ensuring the study’s applicability to real-world conditions.For methylene blue (MB), a concentration of 10 mg L^–1^ is commonly reported in dye-polluted water. Nickel ions (Ni^2+^) at 100 mg L^–1^ represent concentrations observed in electroplating and mining effluents. Chromium species (Cr^3+^ at 1000 mg L^–1^ and Cr^6+^ at 60 mg L^–1^) reflect their higher variability in industrial wastewater. The concentration of silver nanoparticles (AgNP) at 1.68 mg L^–1^ corresponds to their lowest presence in wastewater, considering their application in smaller quantities in antimicrobial and electronic products. The selected concentrations were appropriate for evaluating the performance of the clay-biopolymer microspheres within their expected adsorption limits. These values allowed adequate assessment of adsorption kinetics and capacity without saturation or under-utilization of the adsorbent. We also use these concentrations based on molar absorptivity coefficient (ξ) of each contaminant to minimize possible errors from dilution during adsorption measurements.

### 2.7 Characterization Techniques

The powder X-ray diffraction (XRD) analyses of the solids were conducted with a Miniflex II diffractometer (Rigaku Corporation, Tokyo, Japan), using Cu Kα radiation, λ = 1.54 Å. The angle was varied between 3° and 75°, and all of the samples were processed at a 2°/min rate following the powder method. To obtain the X-ray diffraction patterns, the chitosan and chitosan-poly(vinyl alcohol) microspheres containing kaolinite or saponite were ground into powder using an agate mortar and pestle.

Infrared (FTIR) absorption spectra were acquired with a PerkinElmer FT-IR Frontier spectrometer (Waltham, MA, USA) by using a diffuse reflectance accessory. Specifically, 1 mg of each solid was mixed with 100 mg of KBr and finely pulverized until complete dilution. The pressed samples were analyzed by means of 32 scan acquisitions per spectrum and 1 cm^–1^ nominal resolution.

Scanning electron microscopy (SEM) of the materials was performed with a Vega 3 SBH model EasyProbe digital scanning microscope (Tescan, Brno, Czech Republic). The samples were previously coated with a thin gold layer by evaporation using a Bio-Rad ES100 SEN coating system (Bio-Rad Laboratórios do Brasil, São Paulo, Brazil).

Optical electron microscopy was performed to characterize the microstructure of the solids using a Nicon Y-TV55 Eclipse E100 C-LEDS microscope (Japan). The images were acquired by using the IC Capture V.2.4.633.2555 program.

UV–vis spectra were obtained with a Hewlett-Packard model 8453 UV–vis spectrophotometer (Agilent Technologies, São Paulo, Brazil), using quartz cells with a 10 mm path length.

The cation exchange capacity (CEC) of the clay was calculated by adsorption of methylene blue (MB), which also allowed determination of the specific surface area (SSA) accessible to these molecules. For this purpose, 50 mg of the oven-dried sample was suspended in 10 mL of distilled water, and 0.5 mL aliquots of a 0.1 mol L^–1^ MB solution were added to the suspension with a volumetric buret (2.8 ≤ pH ≤ 3.8). After each addition, the suspension was homogenized by magnetic stirring for 1 min. Then, a small drop was removed from the solution and placed on Fisher filter paper. The fact that nonadsorbed MB formed a permanent blue halo around the suspension aggregate spot on the filter paper meant that MB had replaced cations in the double layer and coated the entire surface. The cation exchange capacity was determined from the amount of MB required to reach the end, according to the following eq 4:
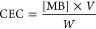
4where CEC is the cation exchange capacity (meq 100 g^–1^); [MB] is the concentration of the methylene blue solution (meq L^–1^); *V* is the volume of the MB solution used during the assay (mL); and *W* (g) is the mass of solid used in the experiment.

The specific surface area accessible to MB (SSA) was calculated according to Hang and Brindley^9^ and Maček et al.^10^ This method assumes that MB molecules cover the particle surface area and that each MB molecule approximates a rectangle with a surface area of 130 Å^2^/molecule. From the amount of adsorbed MB, expressed as CEC (eq 4), SSA was calculated by means of eq 5.
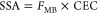
5where SSA is the accessible specific surface area (m^2^ g^–1^); *F*_MB_ is a constant based on the approximate MB area, with a value 7.8043 (m^2^ meq^–1^); and CEC is the cation exchange capacity (meq 100 g^–1^).

### 2.8 Biological Assays

The biological assays were conducted in association with the Mutagenese′s Laboratory to evaluate the viability of applying the microspheres by measuring the acute toxicology to zebrafish (*Danio rerio*) to detect potential biological damage. Finally, the cell viability assay was conducted with the HaCat cell line (immortalized human keratinocyte) to observe the biological viability in solutions treated with the microspheres.

For the acute toxicology and genotoxicity assays involving zebrafish, the experimental protocols were approved by the Ethics Committee on the Use of Animals of the University of Franca (process no. 1606040918) using adult zebrafish (six months old) with a body weight of 0.35 ± 0.18 g and body size of 3.12 ± 0.70 cm; *n* = 147), purchased from a local vendor, and were maintained in stock aquariums with mineral water and aeration for 14 days before the assays.

The acute toxicity test was performed following the OECD 203 guidelines (2019).^11^ The exposure of the animals to the microspheres (I, II, III, IV, V, and VI) was carried out in glass aquariums filled with 2 L of dechlorinated water under constant aeration, without feeding for 96 h. The treatment and analysis procedures were carried out in triplicate using 7 animals/microsphere per repetition, without gender selection and with a negative control.

At the end of the experiment, the surviving fish population was used for evaluation of genotoxicity by the peripheral blood micronucleus test,^12^ where a small drop of blood was collected by caudal puncture and was immediately spread on a clean glass slide, allowed to air-dry, fixed in absolute methanol for 20 min and stained with 10% Giemsa for 10 min. Two slides were prepared per fish. The frequency of micronuclei in erythrocytes was evaluated by scoring 2000 intact cells per fish at 1000× magnification.

Cell viability was assessed using the Resazurin colorimetric method.^13^ Specimens of the HaCat cell line (immortalized human keratinocyte) were seeded in 96-well plates. Each well contained 1 × 10^4^ cells in 100 μL of culture medium (DMEM; Sigma-Aldrich, St. Louis, MO, USA) supplemented with 10% fetal bovine serum (Cultilab, Campinas, SP, Brazil). Twenty-five hours after seeding, the cells were subjected to treatment.

Negative and positive controls (no treatment, 25% dimethyl sulfoxide, DMSO, Sigma-Aldrich, respectively) were used with five chromium solutions (concentrations of 50, 100, 150, 200, and 300 μg mL^–1^) The microspheres were added to the chromium solutions (one unit in the concentrations below 200 μg mL^–1^, two for 200 μg mL^–1^ and four in 300 μg mL^–1^) and the adsorption was read after 24 h at room temperature in the dark.

Afterward, the microspheres were removed from the solutions, the final concentrations were determined, and the solutions were used in the cell culture treatment. After 3 h of treatment at 37 °C, the culture medium was removed and the cells were washed with 100 μL of phosphate-buffered saline (PBS). Subsequently, the cells in each well were exposed to 80 μL of HAM-F10 culture medium without phenol red (Sigma-Aldrich) and 20 μL of resazurin salt (dissolved in PBS). The 96-well plates were incubated at 37 °C for 4 h. Absorbance was measured at 570 nm with a multiplate reader (ELISA-Asys-UVM 340/Microwin 2000) at a reference length of 600 nm. All absorbance results, obtained in the form of cell viability, were calculated and subsequently indicated as IC_50_ (half of the maximum inhibitory concentration). Experiments were performed in triplicate. The results were statistically investigated by GraphPad Prism 6 through an analysis of variance (ANOVA). The means were analyzed using the Tukey test (*p* < 0.05).

## 3 Results and Discussion

**Figure 1:**
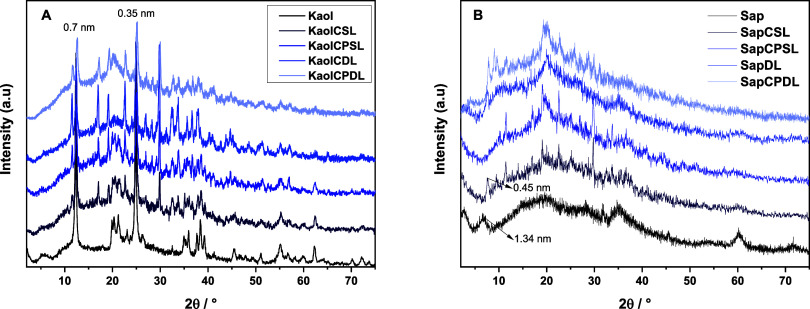
XRD patterns of (A) Kaol and (B) Sap and ground microspheres resulting from the reaction of PVA and chitosan.

The X-ray diffraction patterns (Figure 1) showed that the microspheres were composed of chitosan and PVA, and mixtures of chitosan, PVA, kaolinite, or saponite. The powders obtained from finely grinding the microspheres were also analyzed. For all microspheres obtained from kaolinite and saponite, the pattern displayed a typical broad amorphous halo around 16–28° with a characteristic halo centered at about 21°, indicating the presence of C and PVA biopolymers. However, some differences were observed when kaolinite or synthetic saponite was employed. Kaolinite microspheres presented reflections at 12° with basal spacing of 7.12 Å, which was not affected after synthesis of the microspheres, confirming that the interaction of biopolymers only occurred on the surface and edges of layers. However, when synthetic saponite was employed, the saponite varied from 4.7 to 6.8° to values lower than 3.0°. It was not possible to measure this, confirming the differences associated with the type of clay employed to synthesize the bionanocomposite.

Pure kaolinite exhibits four characteristic peaks within the 2θ range of 20–25°, corresponding to specific crystallographic reflections: (020) at 20° (2θ); (11̅0) at 20.5° (2θ); (111̅) at 21.3°(2θ); and (11̅1) at 23.2° (2θ). These reflections are integral to assessing the structural order of kaolinite, quantified by using the Hinckley Index (HI). The HI is calculated using the formula HI = (*A* + *B*)/Ah, where *A* and *B* represent the intensities at the overlapping regions of the (11̅0) and (111̅) reflections, respectively, and Ah corresponds to the peak intensity of the (11̅0) reflection.^14^ Purified kaolinite presented HI 0.74, evidencing that the kaolinite employed for synthesis was well-ordered kaolinite, signifying minimal stacking faults and greater crystallographic regularity.

The saponite showed three characteristic reflections, assigned to saponite in the PXRD patterns at 2θ = 6.8–7.5° (*d*-spacing of 1.25–1.51 nm), 19.4° (*d*-spacing of 0.47 nm), and 60.5° (*d*-spacing of 0.153 nm) due to the (001), (110), and (060) planes, typical of saponite clay. The XRD patterns of the saponite-based samples displayed two distinct reflections below 10°, which were attributed to the presence of hydrated and nonhydrated cations within the clay’s interlayer space. These reflections highlight the influence of the hydration state of the interlayer cations on the structural organization of saponite. The variations in these reflections were consistent with the changes in the interlayer environment due to the degree of hydration.^14^ This observation further emphasized the dynamic nature of cation exchange and its impact on the clay’s interlayer structure.

It is important to remark that saponite was displaced to values lower than 3°, confirming the presence of biopolymers between saponite layers and evidencing the saponite exfoliation. When TO clay was employed (kaolinite), a conventional composite was obtained. However, when synthetic saponite was employed, an intercalated or exfoliated nanocomposite was formed. The mechanism of intercalation involves the cation exchange of Na^+^ from saponite to the biopolymer, resulting in the separation of individual layers and good dispersion of the inorganic layered matrix around the biopolymer matrix. The amorphous phase, assigned to biopolymers of chitosan and PVA, was common to both microspheres.^15^

The PXRD pattern of chitosan and PVA had an amorphous halo ranging from 10° to 20° corresponding to an amorphous structure.^16^ After cross-linking with kaolinite and saponite, C-PVA/Kaolinite/Saponite also had an amorphous halo, but it was much weaker than before. After copolymer cross-linking with C/PVA, the peaks of kaolinite and saponite, respectively at around 4.7° and 12°, decreased. This reduction in the halo proved that C and PVA were successfully cross-linked with kaolinite and saponite. These results are similar to those described by Cai et al.,^17^ who used alumina and chitosan to synthesize microspheres and observed the same effect via PXRD.

The interaction between C and PVA polymers occurred only on the clay surface since it resembled kaolinite in its purified form, with a restricted basal spacing, making it impossible to insert the polymers directly.

The hydrophilic nature of the polymers led to a decrease in the crystallinity of the bionanocomposites. Additionally, functional groups such as carbonyls and carboxylic acids derived from biopolymers exhibited strong electrostatic interactions with the amine present in C and the hydroxyl present in PVA in the siloxane and aluminol groups on the surface of kaolinite. The single-layer (SL) samples and KaolCPSL structures maintained a crystallinity similar to that of purified kaolinite. However, in the double-layer samples (KaolCDL and KaolCPDL), amorphization was observed due to the dilution effect of kaolinite within the biopolymer matrix.^17^

**Figure 2:**
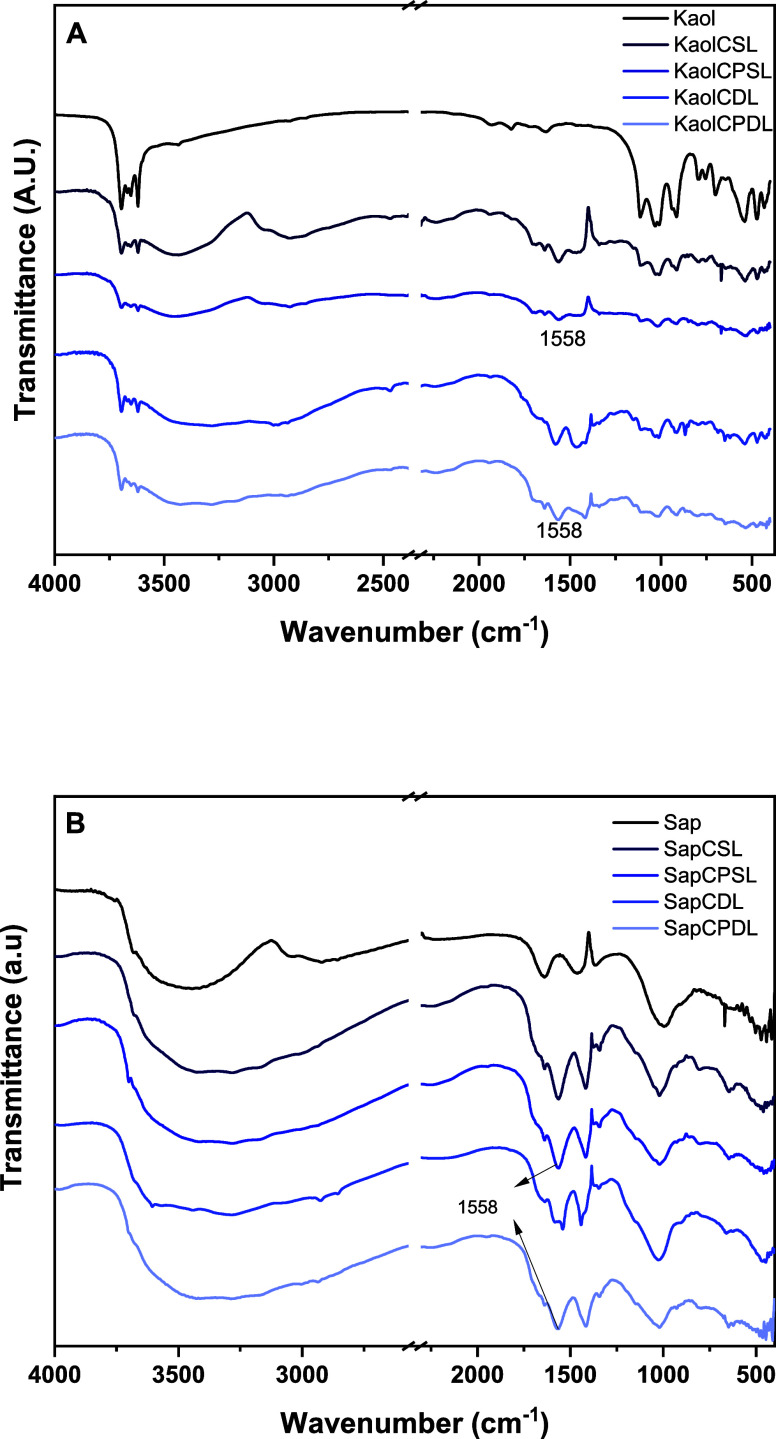
FTIR spectra of (A) Kaol and (B) Sap and ground microspheres resulting from the PVA and chitosan reaction.

Figure 2 displays the infrared absorption spectra of the Kaol and Sap microspheres, respectively. The spectra exhibit typical bands at 3655–3692 cm^–1^, attributed to interlamellar hydroxyl groups (νOH inner surface) and vibrations of the inner surface aluminol (Al–OH) at 938 cm^–1^. The two bands observed at 1500 and 2000 cm^–1^ in the spectrum of unmodified kaolinite (Figure 2A) can be attributed to overtones and combination modes of vibrations associated with the hydroxyl groups present in the kaolinite structure. These features are typical for kaolinite and arise due to the interaction of the Al–OH stretching and bending vibrations with other structural modes. Additionally, minor contributions from adsorbed water or surface hydroxyl interactions could also lead to weak bands in this region. The band at 3620 cm^–1^ corresponds to inner hydroxyl groups (νOH inner). The presence of tactoids and edges of lamellae is confirmed by the 796 cm^–1^ band. Chitosan spectra exhibit a characteristic band at 3448 cm^–1^, related to N–H and O–H. The bands at 2923 and 2853 cm^–1^ are attributed to C–H antisymmetric and symmetric stretching of −CH_2_. The bands at 1654, 1541, and 1384 cm^–1^ are related to amide band I, −NH_2_ bending vibration, and symmetric angular −CH_3_, respectively. PVA displays bands around 3047 and 3170 cm^–1^, assigned to the C–H bonds of PVA. The bands from 3437 to 3441 cm^–1^ represent a strong and broad H-bonded O–H stretching due to the O–H of hydrogen-bonded physisorbed water molecules. The range of 1560–1572 cm^–1^ exhibits bending vibrations of N–H related to amines of the chitosan biopolymer structure.^15^ The difference in intensity between double- and single-layer microspheres is attributed to the increased interaction between the matrix and biopolymers.^18^ The bands show no significant modifications between double and single layers, only variations in intensity in some cases assigned to the copolymers. The 1558 cm^–1^ band is related to the C=N stretching vibration caused by the cross-linking reaction through PVA. After double layer coverage with chitosan and PVA, the intensity of the typical amide bands increased, and the C=N band decreased.^15^ The band at 1200 cm^–1^ attributed to C–O and C–O–C vibration is not present in pure clays (i.e., without the presence of the biopolymers chitosan and poly(vinyl alcohol)). The presence of the C–O–C bond is more intense in the samples where the two biodegradable polymers were used. This may indicate the formation of cross-links between chitosan and poly(vinyl alcohol) that occurs due to the condensation between the OH groups of the alcohol and the OH of chitosan, resulting in the elimination of a water molecule. Additionally only small changes in NH vibrations at 1558 cm^–1^ are observed, corroborating this hypothesis.

Thermogravimetric analysis (TG) and differential scanning calorimetry (DSC) were performed under an oxygen atmosphere to determine the inorganic content of the composite microspheres, investigate their thermal stability, and study the changes induced by cross-linking of chitosan, PVA, and saponite. Table 1 summarizes the thermal parameters.

**Table 1:** Mass Losses of Microspheres Calculated from the TG Curve and Process Attribution

	mass loss (%)		
samples	20–200 °C	200–400 °C	400–900 °C
PVA	4.50	71.70	29.28
C	7.69	57.12	40.67
Kaol	28.78	12.72	16.00
KaolCSL	15.11	24.63	7.96
KaolCPsL	18.44	19.71	11.57
KaolCDL	16.10	22.12	18.55
KaolCPDL	18.61	28.87	24.10
Sap	17.52	5.38	5.80
SapCSL	21.30	29.56	19.01
SapCPSL	23.48	19.40	15.28
SapCDL	16.40	23.83	25.28
SapCPDL	14.33	33.64	23.04

The thermogravimetric analysis (TGA) results, shown in Table 1, indicate the inorganic content remaining after complete thermal degradation at 800 °C. For kaolinite derivatives, the residual inorganic content ranged from 52 to 55% for KaolCSL and KaolCPSL, and from 28 to 40% for KaolCDL and KaolCPDL, reflecting the dilution effect when double-layer coating was applied. Similarly, saponite derivatives displayed residual amounts ranging from 28 to 39% for SapCSL and SapCPSL, and from 23 to 33% for SapCDL and SapCPDL at 400 °C, confirming the effective incorporation of layered synthetic clay into the biopolymeric matrix. The dilution effect was also observed in the double-layer (DL) case.

**Figure 3:**
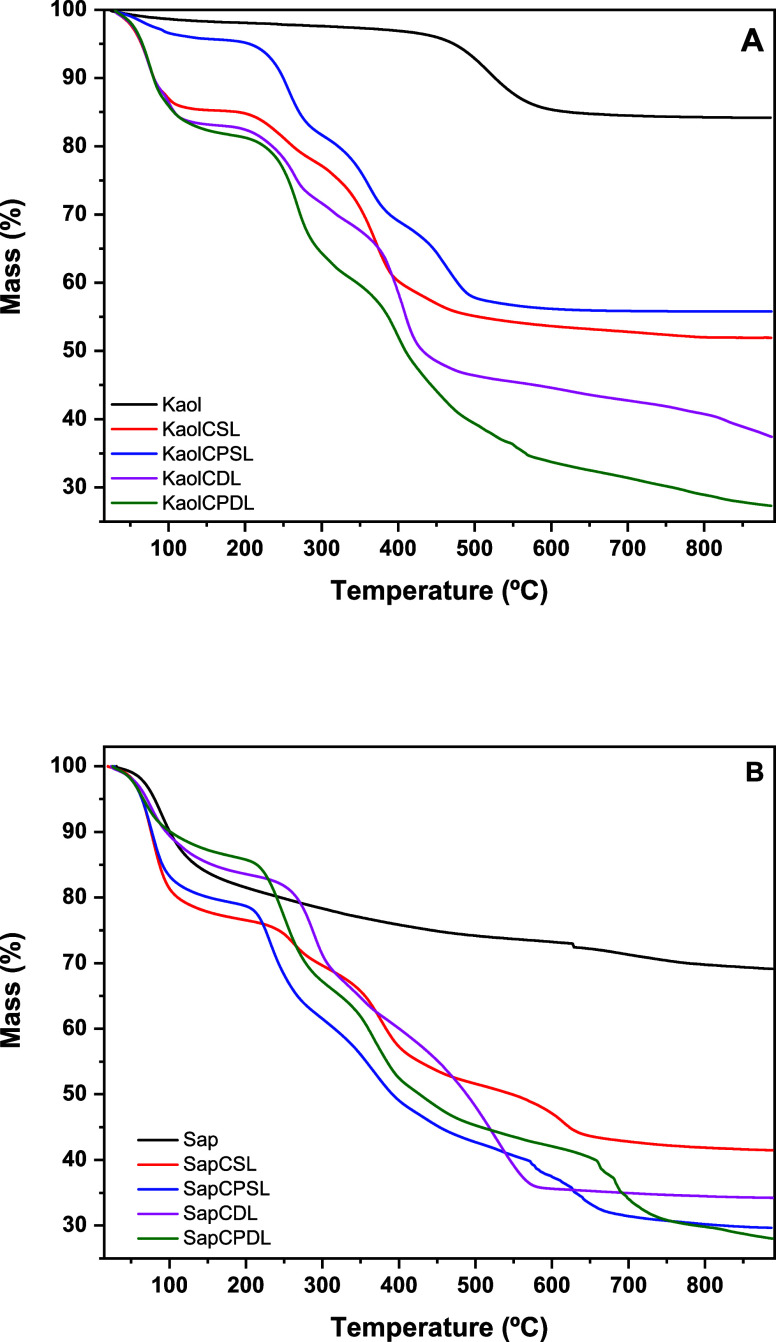
Thermogravimetric curves of (A) Kaol and (B) Sap and microspheres resulting from PVA and chitosan reaction.

Figure 3 shows the TGA curves for the Kaol and Sap microspheres, highlighting the influence of the inorganic matrix content on thermal stability. The reduced inorganic content in saponite derivatives can be attributed to the exfoliation of clay particles, likely induced by water uptake and subsequent leaching of smaller particles during washing. Moreover, the interactions between chitosan and PVA biopolymers with the clay matrices were found to involve electrostatic and hydrogen bonds rather than covalent bonding, as indicated by FTIR analysis.

The degradation behavior of neat chitosan, observed between 198 and 243 °C, was consistent with literature reports. In an oxidative atmosphere, chitosan exhibited strong exothermic effects above 400 °C, signifying efficient oxidation and further decomposition. For the chitosan-PVA cross-linked derivatives, the thermal behavior was similar, with additional decomposition peaks associated with PVA.

Differential scanning calorimetry (DSC) (Figure S7) analysis of the microspheres revealed a decline in the crystallization temperature across all samples, with key peaks assigned to the precursor materials. Kaolinite showed endothermic peaks at 100 °C (water loss) and around 530 °C (dehydroxylation), while saponite exhibited a similar water removal behavior with an additional exothermic peak at 800 °C, attributed to enstatite crystallization. Chitosan displayed water loss at 100 °C and minor exothermic peaks at 300 and 500–600 °C, indicative of crystallization and decomposition, respectively. The differences in thermal stability between kaolinite- and saponite-based nanocomposites were attributed to the cationic nature of saponite, which facilitates better intercalation in the chitosan/PVA matrix, leading to enhanced thermal stability compared to that of the neutral kaolinite-based system.

**Figure 4:**
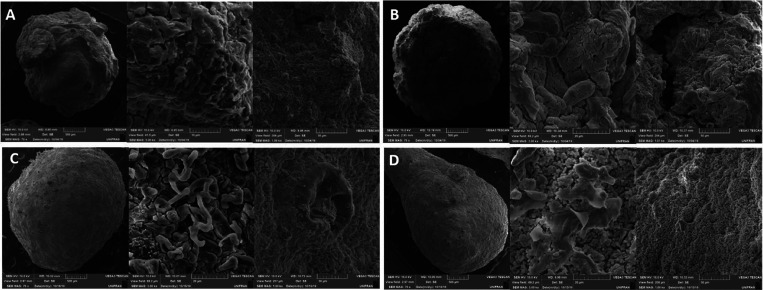
SEM images of samples: (A) KaolCSL; (B) KaolCPSL; (C) KaolCDL; and (D) KaolCPDL. Magnification (70×, 3k×, and 1k×).

**Figure 5:**
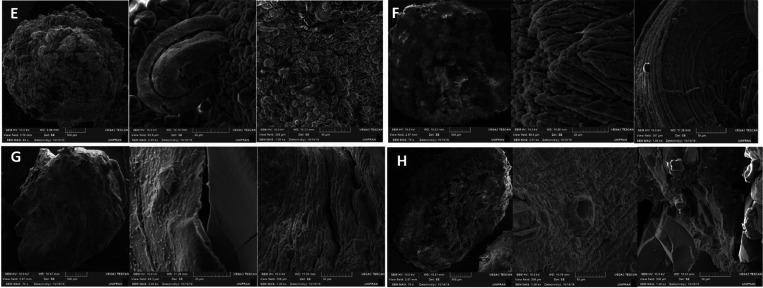
SEM images of samples: (E) SapCSL, (F) SaplCPSL, (G) SapCDL, and (H) SapCPDL. Magnification (70×, 3k×, and 1k×).

The scanning electron microscopy (SEM) images obtained in this study (Figures 4 and 5) provided important information about the microscale morphology of the bionanocomposites. The microspheres had a drop-like shape with small deformations on their surfaces, and some cracks were also present. The clay mineral layers were stacked in a disordered manner. The dispersion of the clay phase in the chitosan (C) matrix and the interface region indicated good phase compatibilization and interaction between the compounds. The samples containing C had greater roughness, resulting in increased specific surface areas and possible adsorption sites. In contrast, the samples formed by a mixture of poly(vinyl alcohol) (PVA) and chitosan had more uniform surfaces with fewer cracks.^19^

The microspheres were synthesized via phase inversion, which could be the reason for the observed cracks and the lack of homogeneity. The high roughness and dispersed regions between the clay and polymers could facilitate the adsorption of metallic ions. Vitali et al. and dos Santos et al.^20,21^ also observed the formation of microspheres with surface deformations and reported good compatibility of clay minerals in C. They also suggested that the regions of fractures and irregularities could improve adsorption.

The pH of the solution plays a critical role in determining the surface characteristics of kaolinite, particularly through the protonation and deprotonation of surface hydroxyl groups at the edges and the permanent negative charges of the silica basal plane. Under acidic conditions, edge hydroxyl groups are predominantly protonated, enhancing positive surface charges, while under alkaline conditions, deprotonation occurs, increasing negative charges. These pH-dependent surface charge variations significantly impact the interactions between kaolinite and the biopolymer matrix during the synthesis process, in turn affecting adsorption, dispersion, and overall microsphere formation. This indicates the importance of pH control to optimize the synthesis conditions.

The experimental method used favors both clay agglomeration due to the absence of a prior clay expansion step. However, based on XRD results, we anticipated that agglomeration is more likely to occur in kaolinite particles, given their neutral charge. In contrast, saponite microspheres exhibit a more homogeneous dispersion due to their cationic nature. This observation is corroborated by the SEM images, which reveal uniformly distributed microspheres at various magnifications as well as by adsorption experiments employing optical microscopy. For kaolinite microspheres, agglomerated clay platelets show localized adsorption of the MB dye at specific points. Conversely, this effect is not observed in saponite samples, where the dispersion remains consistent and uniform.^22−24^

In the microspheres containing the kaolinite matrix and saponite, there were only slight increases in CEC and SS between the samples with single and double layers. This may have been because kaolinite is a neutral clay mineral with a low CEC of only 15 mmol 100 g^–1^. As a result, interactions only occur between polymeric compounds. Microspheres containing only C had higher CEC and SS, indicating that the addition of PVA directly affected the porosity of the microspheres. PVA has a strong electrostatic interaction between the hydroxyls in its structure and the C-free amines, reducing both the cation exchange capacity and the specific surface area of the microspheres.^25^ This reduction in specific surface area can lead to a decrease in the available adsorption sites on the microspheres’ surface, ultimately affecting their adsorption capacity.

In the microspheres containing saponite, significant reductions in the CEC and SS concentrations were observed. This may have been due to charge compensation, since trioctahedral substitution in trivalent saponite by divalent cations generates an excessive positive charge of the layers. Saponite has a higher CEC of 100 mmol 100 g^–1^, which could also have affected the observed behavior. Additionally, the interaction of organic portions may occur more easily on the higher specific surface area of saponite.^26,27^ We also observed that a higher concentration of chitosan resulted in the formation of a smaller pore size and a more restricted network in the porous C microspheres, decreasing the number of possible adsorption sites of the double-layer microspheres and reducing the specific surface area. The observed reduction in the specific surface area reveals the importance of careful design and optimization of microsphere fabrication processes to achieve the desired properties and performance.

The specific surface area (SSA) and cation exchange capacity (CEC) measurements were assessed using methylene blue (MB) as a probe (Table 2), primarily reflecting the materials’ capacity to adsorb cations. For unmodified saponite, the high SSA observed is attributed to the exchangeability of interlayer cations with MB molecules. In contrast, the reduced SSA of chitosan-modified saponite (SapCSL) is due to the polymer coating, which restricts access of MB to the interlayer sites. This result highlights the barrier effect of the chitosan matrix, which limits the availability of exchangeable sites and influences the adsorption behavior of the modified material.

**Table 2:** Summary of CEC and SS Results of the Samples

sample	CTC (meq 100 g^–1^)	SS (m^2^ g^–1^)
Kaol	4.00	31.22
KaolCSL	7.04	54.96
KaolCPSL	3.51	27.36
KaolCDL	8.24	64.34
KaolCPDL	4.98	38.85
Sap	26.13	203.93
SapCSL	1.52	11.86
SapCPSL	3.15	24.59
SapCDL	2.28	17.79
SapCPDL	3.94	30.74

The water uptake percentage of the samples was analyzed to determine the swelling behavior. Factors such as the pore size, surface roughness, and pore structure can directly affect the water absorption rate. The polymers used in this study have hydrophilic characteristics, and the clay minerals kaolinite and saponite are neutral and cationic respectively, which can affect the swelling behavior of the synthesized microspheres.^28^

**Figure 6:**
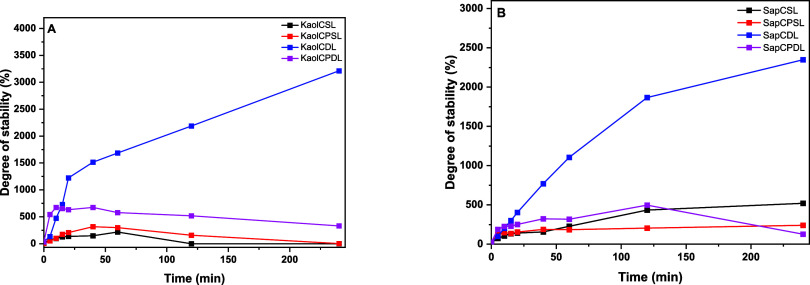
Kinetic study results of water uptake during acid stability testing of microspheres composed of (A) Kaol and (B) Sap.

Figure 6 shows the maximum swelling values of the KaolCSL and KaolCPSL samples after 120 min. The lower water sorption values for the KaolCSL composite are due to the intercalation of the polymer in the clay mineral galleries, which reduces the water uptake capacity of both materials. According to ref (29), the crystalline material (purified kaolinite) inhibits the diffusion of water, and the crystallinity of composites increases with a reduction in swelling. The complete swelling of KaolCDL took 60 min, while KaolCPDL took 120 min, and the absorptive equilibrium time was 200 min for purified kaolinite microspheres. The greatest degree of swelling occurred in the KaolCPDL microspheres since the inorganic matrix was fully diluted and neutral, thus not interfering with the water absorption process. Higher concentrations of the polymeric portion increase the swelling.

The SapCDL microspheres had higher swelling percentages due to the greater concentrations of C in their composition. Although Sap has swelling behavior, charge changes can delay the absorption of water molecules, and the equilibrium time for water accommodation for samples containing Sap had variable behavior. Single-layer microspheres had a lower degree of swelling than did double-layer samples. However, the insertion of PVA increased the swelling degree by providing greater stability and control of the swelling of the matrix.

Overall, the adsorptive properties depend on the PVA mass fraction and the water activity. The good compatibility of the components, resulting from the presence of hydrogen bonds between specific groups (hydroxyl, amide) of the C and PVA chains, can cause an increase in the molecules’ size and therefore a decrease in the pore volume and water diffusion rate. In the interaction studies between PVA and C carried out by Mucha and Kawinska,^30^ the C sample had more acetamide groups than the other samples and absorbed much more water. We also observed that double-layered microspheres had a greater swelling capacity. In microspheres containing C and PVA, a strong reduction in their absorption capacity was observed and the higher contents of PVA in the mixtures were associated with smaller amounts of water uptake. It is noteworthy that PVA is a hydrophilic polymer, meaning that it has a strong affinity for water molecules. When it is added to chitosan/clay nanocomposites, it can directly affect the porosity and swelling behavior of the microspheres. This is because PVA has a strong electrostatic interaction between the hydroxyl groups in its structure and the C-free amines in chitosan, which reduces both the cation exchange capacity and the specific surface area of the microspheres. As a result, the pore volume is decreased, and the water diffusion rate is slower, leading to a reduction in water uptake capacity. The higher the content of PVA in the mixture, the smaller the amount of water that can be absorbed by the microspheres.

**Figure 7:**
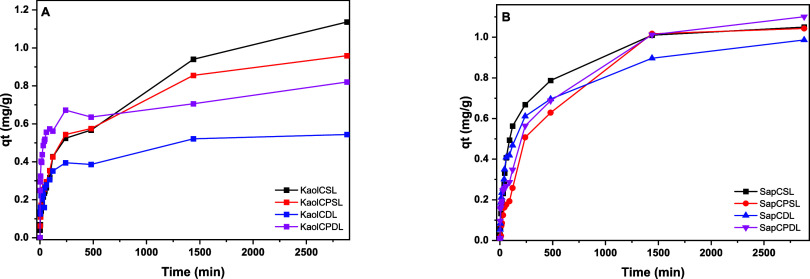
Adsorption kinetics (condition for MB: *C*_0_ = 10 mg L^–1^, *t* = 0–2880 min, using as adsorbate and 100.0 mg of each microsphere as adsorbent. (A) Kaol and (B) Sap).

Figure S5 shows the results of the swelling index (Kit) obtained through a gravimetric evaluation. The data indicate that the double-layer C microspheres had a high swelling index due to the high solubility of C at acidic pH, which promotes swelling. It also suggests that the presence of free amino groups in the C structure contributed to the swelling behavior observed in the double-layer microspheres composed only of C. The double-layer microspheres containing PVA exhibited a mass loss over time, starting from 120 min, suggesting that the electrostatic interaction between C and clay minerals can be easily disrupted by the presence of H^+^ ions. These results are consistent with the results of the stability tests obtained via transmittance shown in Figures 7 and S8.

The transmittance stability test associates the lack of stability of the hybrid microspheres with their dissolution, which can result in the suspension of biopolymer fragments and clay mineral particles, thus, reducing the percentage of transmittance. The turbidity can also increase due to the dilution of other components present in the microspheres. However, because of the quick and easy dissolution of chitosan in an acidic medium, it is difficult to measure the exact amount of adsorption or coordination capacity accurately. The results of the referenced work, however, did not follow the acid stability kinetics and the analysis of the degree of swelling of the microspheres according to the masses.^31^

In all the experiments performed (Figure S9), the microspheres dissolved more slowly due to the lesser contact between the acid and C.^32^ The clay mineral fraction and PVA indicate that the amine groups formed hydrogen bonds or were condensed to the hydroxyl groups in the case of the copolymer with PVA. The microspheres composed of the clay mineral Sap had greater stability as a function of time in acidic solution, indicating the existence of electrostatic interactions between the ammonium cations (NH^4+^) formed from the amino groups (R-NH_2_), which are present in the C structure, and is also associated with the fact that clay minerals have a high cation exchange capacity. Therefore, saponite made these interactions more effective, resulting in an intercalated nanocomposite in which the biopolymer matrix promoted the effective separation of the lamellae, further increasing the contact surface between the organic and inorganic phases and resulting in greater stability of this class of microspheres.

Microspheres containing only C in double layers had similar behavior, with a high swelling degree under acidic conditions. This fact can be attributed to the increased concentration of C. In the transmittance analysis, the behavior was directly influenced by the type of clay mineral used. KaolCDL remained stable for 40 min, while SapCDL remained stable for 120 min, because when it came into contact with the acidic aqueous solution, there was rapid exchange of cations and possible formation of bonds between the amine and hydroxyl compounds present in Sap in the interlamellar space of the clay mineral in the double-layer C microspheres.

### 3.1 Kinetic Adsorption of Contaminants MB, Ni^2+^, Cr^3+^_,_Cr^6+^, and AgNP

The use of two different types of lamellar matrices shed light on the difference in adsorptive behavior, enabling analysis of the ionic coordination of metal ions both in the clay mineral structure and in the functional groups present in the biopolymers (R-NH_2_ and R-OH). Adsorption can occur at the superficial aluminol and silanol sites.^33^

**Figure 8:**
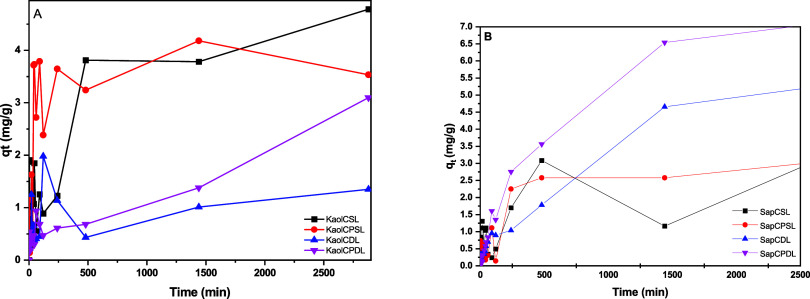
Adsorption kinetics (condition for Ni^2+^: *C*_0_ = 100 mg L^–1^, *t* = 0–2880 min, used as adsorbate and 100.0 mg of each microsphere as adsorbent: (A) Kaol and (B) Sap).

Staining with methylene blue (MB) dye is applied to evaluate the adsorption capacity of TO and TOT clay minerals (Figure 8). MB interacts with kaolinite due to surface impurities since it has no charges. MB is a cationic dye, meaning that it has a positive charge. Its interaction with saponite occurs mainly through electrostatic forces, where the negative charge on the saponite surface attracts and retains the positive ions of MB. The adsorption capacity of a clay is directly related to the negative charge on the lamellar surface. In this case, it was much more pronounced for synthetic saponite clay. This negative charge can be neutralized by the adsorption of positively charged cations such as MB dye.^34^

Dong et al.^35^ synthesized C microspheres for adsorption of methyl orange dye, a test carried out in a microfluidic column where the continuous process allowed the efficient adsorption of the evaluated adsorbate, thus showing the importance of studying microspheres containing C and inorganic materials to reduce the cost of industrial operations. All microspheres had an optimal time of 2880 min. Hence, the gradual adsorption may have been associated with the fact that the process occurred statically without forcing contact between the microspheres and the molecules of the adsorbate.

The single-layer composite systems showed a greater sorbent capacity. In general, the hybrid microspheres showed a gradual increase in qt up to 500 min, and it remained constant up to 1500 min for both Kaol and Sap (Figure 7). The adsorptive capacity observed in the kinetic study was similar between groups (single-layer and double-layer kaol). Between the KaolCDL and KaolCPDL samples, the qt values found at the equilibrium time were 0.54 and 0.84 mg g^–1^, respectively. For samples SapCDL and SapCPDL the values found were 0.98 and 1.1 mg g ^–1^ respectively. Thus, it is possible to infer that PVA acted directly in the adsorptive process, contributing to the generation of new adsorption sites due to the presence of hydroxyl groups in its structure. This effect observed for the neutral clay mineral was less pronounced for the microspheres based on saponite since for this class of microspheres the high cation exchange capacity significantly contributed to the adsorptive process, facilitating the retention of cations in the hybrid structure. Therefore, great differences were not observed in the kinetic profile, as well as in the values of maximum adsorption capacity as a function of time.

With regard to the images taken after the MB adsorption process,^36^ the existence of saturated points mainly in the SapCPSL microspheres can be explained by the fact that adsorption acted on the surface of the microspheres. At the beginning, the process of MB formation on the microsphere surface occurred quickly at the NH adsorption sites of chitosan. The microspheres provided sites for adsorbing MB. Then, the dye infiltrated the microspheres. The number of channels and pores in the microspheres determined the infiltration rate as well as the adsorption rate in the later stage since the initial concentration and temperature of the solution were fixed so the porous microspheres could achieve adsorption equilibrium quickly. This can be clearly observed at 8 h in both kinetic experiments, involving neutral clay (kaolinite) compared to cationic clay (saponite). The amount adsorbed varied between 0.4 and 0.6 mg g^–1^ in the case of kaolinite and ranged from 0.6 to 0.8 mg g^–1^ in the case of saponite. This effect can be explained by the greater water uptake of the cationic clay, inducing swelling of the clay layers and correlating the MB uptake. Saponite contains more sites for adsorption, so the presence of microspheres containing synthetic Sap makes it possible to predict and standardize the structural configuration of the material while maintaining greater chemical and thermal stability in the adsorption tests. The images show the efficiency in the adsorption process after the kinetic study of the adsorption of the MB dye on the hybrid microspheres (Figures S10 and S11).

The high roughness and large dispersed regions between the clay and polymer components play a crucial role in facilitating the adsorption of metallic ions. These regions provide additional surface areas and specific adsorption sites, enhancing the interaction between the nanocomposite and metal ions. The microstructure of the clay–polymer matrix was carefully examined by optical microscopy, and specific adsorption sites were identified, as indicated in the updated figure. These features are keys to understanding the mechanisms behind the efficient metal ion uptake and the overall performance of the nanocomposite in adsorption applications. The cationic saponite clay is noteworthy regarding the favorable and homogeneous dye distribution on all of the microspheres. However, the microspheres based on kaolinite exhibited a heterogeneous dye distribution, revealing the formation of some agglomerates. Additionally, kaolinite had a lower specific surface area (near 15 m^2^ g^–1^) and neutral surface charge (principally without isomorphic substitution). This result indicated the effect of cationic or neutral clay in microspheres on tailoring the selectivity of adsorption at the surfaces.

Molecular absorption spectroscopy in the ultraviolet–visible region (UV–vis spectroscopy) is an important tool to analyze the concentration of compounds in aqueous media and the behavior of the chromophore groups of organic substances. The spectra in Figure S4 (Supporting Information) reveal the ideal concentration for the complexation of Ni^2+^ ions. The optimal concentration was defined according to the selective detection of nickel ions. Based on this study, the trace metal concentration was then varied to obtain the calibration curve.

When we used Ni^2+^ as an adsorbate (Figure 8), the highest adsorptive capacity occurred for the SapCPDL sample (7.21 mg g^–1^). This can be attributed to the structure of the hybrid microspheres obtained. The association of the cationic nature of the clay mineral and, the presence of functional groups from both C and PVA, favored the interaction with Ni^2+^ metallic ions.^37^ In this regard, Ghaee et al.^38^ obtained a qt value of 5.21 mg g^–1^ for silica/chitosan samples.

Findon et al.^39^ suggested that nickel ions can be chelated together with NH_2_ and OH groups in the C chain. In turn, Chui et al.^40^ confirmed that C amino acid groups are the main effective binding sites of metal ions, forming stable complexes via coordinated bonding. The free electrons present in nitrogen can establish coordinated bonds with transition metal ions, according to affinity and polarizability, as classified by Pearson’s principle. Some hydroxyl groups present in these biopolymers can also act as donors. Thus, deprotonated hydroxyl groups may be involved in the coordination of metallic ions. Inoue et al.^41^ suggested that C forms chelates with metallic ions, releasing H^+^ ions and indicating the formation of a complex between C and Ni^2+^ ions.

The adsorption mechanism of microspheres depends on the available sites, specific surface area, ion exchange capacity, electrostatic interactions, hydrogen bonds, and van der Waals forces. The standardization of the saponite structure and its swelling rate demonstrated great applicability for the adsorption of different types of contaminants. The modification and insertion of polymeric components increased the adsorption rate, aiming at the regeneration and reuse of these materials.^42^

Materials containing saponite had improved results due to their swelling and surface charge characteristics. The greatest amount of Ni^2+^ was found for SapCPDL, 7.0 mg g^–1^ at 48 h, while solid KaolCSL had 4.5 mg g^–1^ at the same time. This result confirms the role of neutral and cationic clay in adsorption of metals, in addition to having a similar morphology. The microspheres containing SL presented more satisfactory results in relation to DL due to the greater number of available adsorption sites. The ease of producing SL contributed to our choice of microspheres for subsequent studies.

**Figure 9:**
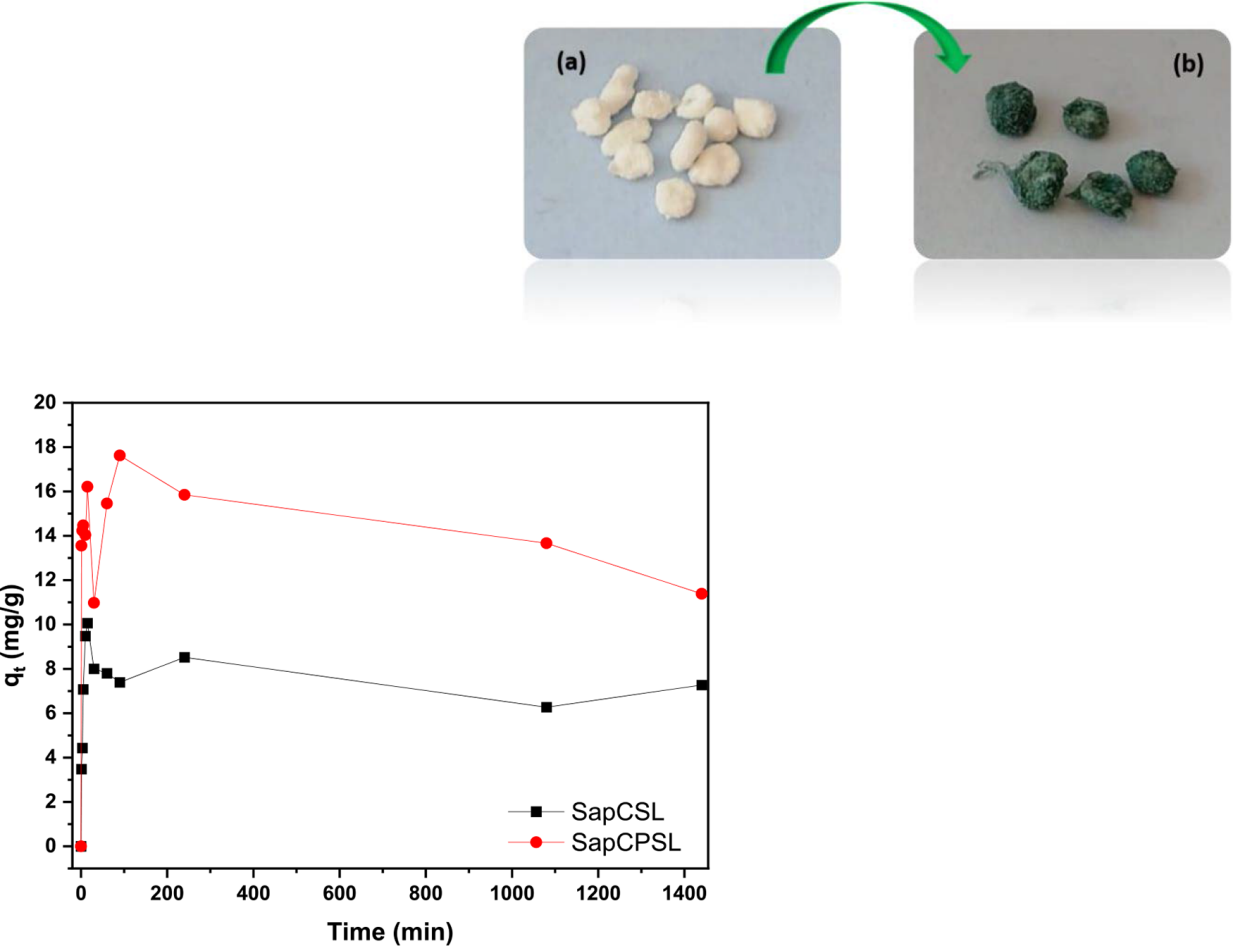
Adsorption kinetics (condition for Cr^3+^: *C*_0_ = 1000 mg L^–1^, *t* = 0–2880 min, used as adsorbate and 100.0 mg of each microsphere as adsorbent) and image of the bionanocomposites: (a) SapCSL and SapCPSL before the Cr^3+^ adsorption process and (b) after that process.

Figure 9 depicts the adsorption capacity of the SapCSL and SapCPSL bionanocomposites in relation to Cr^3+^ metal ions at 1000 mg L^–1^. The graph shows that the maximum adsorption capacity of SapCSL occurred in the first minutes of contact, while the maximum adsorption capacity of SapCPSL was approximately 1800 mg g^–1^ after 30 min of contact.

Wan Ngah et al.^43^ reported that unmodified C exhibited low adsorption capacity when adding PVA and significantly increased the acid stability of C. By increasing the pH, the adsorption efficiency of Cu^2+^ ions increased in C/PVA. Bavaresco et al.^44^ evaluated the variation of maximum adsorption capacity of Cr^3+^ in clay mineral samples according to the pH of Cr^3+^ nitrate solutions adjusted to a pH range of 4.5–5.5, highlighting that the Cr adsorption capacity increased with rising pH of the clay mineral due to more charges on the adsorption surfaces. This negative charge can be neutralized by the adsorption of positively charged cations, such as metal ions.^34^

Wan Ngah et al.^45^ studied the removal of Cr^3+^ and Cr^6+^ from an aqueous solution and found that the removal of Cr^6+^ was difficult, making it the most toxic form of chromium, even at low concentrations. The authors reported that the adsorption mechanism occurred based on parameters such as the amount of functional groups available, degree of ionization, pH and concentration of the adsorbate.

Findon et al.^39^ suggested that metal ions can be chelated together with the NH_2_ and OH groups in the C chain. Chui et al.^40^ confirmed that the amino acid groups of chitosan are the main effective binding sites for metal ions, forming stable complexes via coordinated binding. The free electrons present in nitrogen can establish coordinated bonds with transition metal ions, according to affinity and polarizability, as classified by Pearson’s principle. Some hydroxyl groups present in these biopolymers can also act as donors. Thus, deprotonated hydroxyl groups may be involved in the coordination of metal ions. Inoue^41^ suggested that chitosan forms chelates with metal ions, releasing H^+^ ions, suggesting the formation of a complex between chitosan and Cr^3+^ and Cr^6+^ ions. The exceptional adsorption capacity of SapCPSL for Cr^3+^ ions (17.63 mg/g) can be attributed to the material’s strong chemical compatibility with Cr^3+^, a hard acid, and the hydroxyl and amine groups in the chitosan-PVA polymer matrix, which act as hard bases, as described by the Pearson Hard and Soft Acid–Base (HSAB) principle. Furthermore, the synthetic saponite provides exchangeable interlayer cations, enhancing ion exchange and adsorption.

Figure S12 presents data related to the adsorption kinetics of Cr^6+^ ions by the SapCSL and SapCPSL bionanocomposites. The SapCPSL bionanocomposite had the greatest adsorption capacity, of around 20 mg g^–1^, after 120 min of contact.

The quantification of Cr^6+^ in an aqueous solution is carried out by UV–vis spectrophotometry based on the yellow color of the chromate ions, with the maximum absorption occurring at a wavelength of 350 nm and with the linear range varying from 0.5 to 100 mg L^–1^ of Cr^6+^, thus justifying the concentration used in this study.^46^ Buerge and Hug^47^ also used the direct analysis method to detect Fe^2+^ and Cr^6+^ concentrations, explained by its speed and reliability in contrast to the DPC method, which requires prolonged preparation of reagents and produces a color complex with limited stability.

The pH value of the aqueous ion solution is generally the key parameter for the adsorption process and can strongly affect the removal of ions from aqueous solutions since it controls the surface charge of the bionanocomposite adsorbent and the chemical nature of the metal cations. In acidic conditions, the predominant chromium species were HCrO^4–^ and Cr_2_O7^2–^ in aqueous solutions, and the surfaces of the adsorbents were highly protonated, which favored the uptake of Cr^6+^ in the anionic form.^48,49^

The reduction of potassium dichromate (K_2_Cr_2_O_7_) occurs when the chromium dichromate ions (Cr_2_O_7_^2–^) react with carbon, possibly from the chitosan structure, promoting the oxidation of carbon and undergoing reduction. In solution, it may undergo a change in pH. Therefore, as the pH increases, the potassium dichromate solution tends to become less concentrated and can eventually precipitate as a chromic species, which tends to be yellowish-green in color. However, according to Mahmood et al., there are different selection methods, relating the reduction of Cr^6+^ in its trivalent form (Cr^3+^) using sodium metabisulfite (Na_2_S_2_O_5_) and ferrous sulfate (FeSO_4_), and then precipitating Cr^3+^ as hydroxide using precipitating agents such as Ca(OH)_2_, NaOH or a combination of both. Our results corroborate this finding about the phase inversion method for the synthesis of microspheres using NaOH. Even after washing, the same material may be released into the absorption solution, thus changing the pH and consequently precipitating and reducing hexavalent chromium to its trivalent form.^50^

For AgNP adsorption, both SapCSL and SapCPSL (Supporting Information Figure S13) showed efficiency within 15 min, with SapCPSL demonstrating a further increase in adsorption after 120 min, generating excess agglomerates, which in turn precipitated due to ion exchange arising from the surface of the microspheres. The ionic interaction may have occurred due to the change in pH in the medium. Studies have described the use of chitosan/AgNP matrices as antimicrobial agents that can effectively prevent bacterial invasion and inhibit the growth of pathogenic microorganisms. This is a preliminary alternative for the reuse of AgNP-enriched microspheres from this study.^51,52^

Table 3 presents the adsorption performance of chitosan/clay composite materials for various contaminants, highlighting key parameters such as the adsorption capacity, removal efficiency, and experimental conditions. The data summarize the interactions between the adsorbents and different pollutants, including heavy metals, organic dyes, and emerging contaminants, under varying pH, temperature, and contact time conditions.

**Table 3:** Summarized Data about the Adsorption of Chitosan and Chitosan/Clay Solids Applied to the Removal of Various Contaminants

material	pollutant	initial concentration (mg/L)	time (min)	*q*_t_ (mg/g)	reference
chitosan-PVA beads	Cr(VI)		180	3.5	Pal et al.^53^
chitosan-PVA beads	Cu(II)	0	1890	4	Pal et al.^53^
chitosan-MMT	congo red			53.42	Pal et al.^53^
chitosan-MMT	Cr(VI)	60	240–360	41.67	Wang et al.^54^
chitosan-bentonite	Cr(VI)		30	22.17	Biswas et al.^55^
chitosan-vermiculite	sunset yellow FCF	500	1440	175.1	Biswas et al.^55^
chitosan-bentonite	Ni(II)	200		12.35	Biswas et al.^55^
chitosan-kaolinite	Cu(II)	250		116.22	Et-Tanteny et al.^56^
chitosan-kaolinite	Cd(II)	250		147.64	Et-Tanteny et al.^56^
Kaol-CSL	MB	10	2880	1.1	this work
Kaol-CDL	MB	10	2880	0.45	this work
Kaol-CPSL	MB	10	2880	0.9	this work
Kaol-CPDL	MB	10	2880	0.75	this work
Sap-CSL	MB	10	2880	1.0	this work
Sap-CDL	MB	10	2880	0.9	this work
Sap-CPSL	MB	10	2880	1.0	this work
Sap-CPDL	MB	10	2880	1.1	this work
Kaol-CSL	Ni(II)	100	2880	4.79	this work
Kaol-CDL	Ni(II)	100	2880	3.10	this work
Kaol-CPSL	Ni(II)	100	2880	3.51	this work
Kaol-CPDL	Ni(II)	100	2880	1.36	this work
Sap-CSL	Ni(II)	100	2880	3.52	this work
Sap-CDL	Ni(II)	100	2880	4.0	this work
Sap-CPSL	Ni(II)	100	2880	3.13	this work
Sap-CPDL	Ni(II)	100	2880	7.20	this work
Sap-CSL	Cr(III)	1000	1440	7.95	this work
Sap-CPSL	Cr(III)	1000	1440	17.6	this work
Sap-CSL	Cr(VI)	60	1440	12.5	this work
Sap-CPSL	Cr(VI)	60	1440	4.8	this work
Sap-CSL	AgNP	1.68	1000	0.02	this work
Sap-CPSL	AgNP	1.68	1000	0.9	this work

### 3.2 Kinetic Modeling

Tables S5 and S6 present the experimental data of the adsorption of MB dye and Ni^2+^ ions by the microspheres together with the curves calculated from the four kinetic models tested.

In general, it appears that the intraparticle diffusion and Elovich models best fit the experimental data, indicated by the low value of *X*^2^ (<1), meaning that the intraparticle diffusion and Elovich models describe the kinetic mechanism of the adsorption of MB and Ni^2+^. This was also explained by the fact that the correlation coefficient (*R*^2^) was near 1 in some cases.

From the Elovich model, it is possible to conclude that the microspheres presented a value of parameter α smaller than parameter β, indicating that the adsorption rate is much lower than the desorption rate. This also shows that these materials can hinder the diffusion of the adsorbate throughout the matrix, suggesting that clay mineral particles are well dispersed throughout the matrix, giving these microspheres barrier properties by, hindering the adsorption process in the active sites available in the structure of clay minerals

It is important to note that parameter β is a constant related to the extension of surface coverage and can actually decrease with the increase in the initial concentration of the adsorbate. Probably the polymer matrix affects the cation exchange capacity of the microspheres, and the adsorption occurs by a synergistic effect between the clay minerals and biopolymers.

According to the intraparticle diffusion model, the removal mechanism occurs exclusively at the external sites of an adsorbent with low porosity. Thus, the absorption rate and the value of parameter k must vary reciprocally with the first power of the diameter for a given mass of adsorbent. This inverse relationship also holds for porous adsorbents when the transport rate to internal surface areas is controlled by external resistance.^57^ In some synthesized hybrid microspheres, the intraparticle diffusion model also fits very well, with low values of *x*^2^ and correlation coefficients close to 1, which confirms that a synergistic effect of clay minerals and biopolymers controls the adsorbate adsorption rates. Obviously, the very slow adsorption rates are due to the evaluated experimental conditions (at rest). The experimental data confirm that Sap, Kaol and chitosan were very well dispersed, and their barrier properties could hinder the adsorption of MB and Ni^2+^. Probably the MB and Ni^2+^ mechanisms involve the cation exchange capacity of Sap dispersed in the polymer matrix, and the main adsorption mechanisms are affected by the polymer intercalation between the Sap layers.

The microspheres behaved differently from reports in the literature regarding contact time. Anna et al.^58^ reported that increasing contact time did not result in increased metal ions adsorbed on bentonite (powder). During the initial sorption phase, a large number of sites on the surface are available for adsorption. However, for the hybrid microspheres evaluated in this study, the contact time was a primordial parameter to increase the adsorptive capacity, demonstrating the type of interaction between the adsorbent and adsorbate proposed by the Elovich model.

We found that the pseudo-first order kinetic model suffered inadequacies when applied to the adsorption of potentially toxic metals using the synthesized microspheres. The experimental qe values differed from the theoretical values. This can be explained by the low linearity of the plots obtained from this study and the discrepancies observed in the theoretical and experimental values of chemisorption.^59^

If the adsorption of potentially toxic metals by the microspheres aligns with the Elovich model, this suggests that the initial interaction stability between the Sap-containing microspheres and the polymer plays a significant role in the adsorption process. The Elovich model, often used to describe chemisorption on heterogeneous surfaces, implies that the rate of metal adsorption decreases over time due to active site saturation, reflecting the complex surface interactions within the microsphere structure in the liquid phase. The adsorption processes of these materials can occur in stages, where the first one involves the surface interaction of metallic ions. Then, if the surface interaction does not occur, the metallic ions can settle between the layers, followed by a desorption process. This behavior was reported by Oladoja et al.^60^ In the initial model, the concentration was increasing, while the desorption constant, β, was decreasing. In other words, adsorption was increasing. At lower concentrations, many other ions with higher adsorption energy can compete and desorb the metal ions, while at higher concentration, the number of metal ions for adsorption is also higher, so the desorption decreases.

The correlation coefficients obtained were almost linear, which showed that the Elovich model fitted the data well. The model provided a good correlation for adsorption on surfaces, such as microspheres. Furthermore, it also showed that along with surface adsorption, chemisorption was also a dominant phenomenon.

The Elovich equation was also used to interpret the adsorption kinetics of Ni^2+^ on clays by Sen and Bhattacharyya.^61^ This equation was found to be useful mainly to describe the chemical adsorption that occurs in SapCSL for trivalent chromium ions specifically. In other cases, the adsorption of trivalent and hexavalent chromium ions best fitted the pseudo-first order and pseudo-second order equations respectively. In turn, the activity of the silver nanoparticles was best described by the pseudo-first order model.

The Elovich equation has also been used to interpret the adsorption kinetics of Ni^2+^ on clays.^61^ This equation was considered useful mainly to describe chemical adsorption on highly heterogeneous adsorbents, but no defined mechanism for adsorbent-adsorbent interaction could be suggested,^62,63^ thus providing a large surface area for interaction. Similar results have also been reported by ref (64) for the adsorption of Cu^2+^ on peat.

The pseudo-second order model, only has limited applicability to the adsorption of Ni^2+^ on clays according to Sen and Bhattacharyya.^61^ Interactions can occur directly with the polymeric fraction of the microspheres.

The interaction kinetics of Ni^2+^ and MB may have occurred due to the contributions of all four mechanisms determining the adsorption process.^65^

### 3.3 Enhanced Colorimetric Sensing Study

Hybrid arrays of colorimetric sensors do not require sophisticated equipment and can produce results through color changes. This method has proved to be extremely effective to identify and quantify aqueous phase analytes for environmental monitoring.^8^ The adsorption behavior of chitosan can enhance the sensing efficiency in the capture of metallic ions in an aqueous medium since the detection is based on the intermolecular interaction of the compound present in the environment based on the type of analyte detection.

Fukushima and Aikawa^66^ investigated the insertion of (XO) with anionic characteristic interacted with the cationic polymer poly(diallyldimethylammonium chloride) (PDADMAC) by electrostatic affinity. XO can be composed of six negative charges, namely, a sulfonate group, four carboxylates, and a phenolate group. The electrostatic interaction of XO with the polymer can occur through negatively charged XO groups. The results showed that the sensor was selective for certain metallic ions.

Our preliminary tests showed that the matrices containing saponite and chitosan exhibited significant changes in color due to the presence of different metallic ions. Figure S14 refers to the preliminary test with metallic ions (Cu^2+^, Ni^2+^, Cr^3+^, Co^2+^ and Cr^6+^), The system appears pink when not complexed (pink microsphere), and the coordination with the ions produces an instantaneous change in color. No leaching of XO was detected using either sample, which confirms that microspheres could act as very interesting colorimetric markers of enhanced metal detection for environmental quantification purposes.

The addition of Ni^2+^, Cr^3+^, Cr^6+^, Cu^2+^, and Co^2+^ + XO in the presence of C affected the absorption spectrum pattern as well as the color of the microspheres.

An XO solution (300 mg L^–1^) was prepared where the microspheres remained under swelling for 200 min. After this process, the weighed amount (0.10 g) of sorbents modified with XO (SapCPSL) was deposited in solutions of 1000 mg L^–1^ of metal ions (Cu^2+^, Ni^2+^, Cr^3+^, Co^2+^ or Cr^6+^), then left at rest for 24 h. The aliquots were analyzed via UV–vis spectrometry, and the microspheres with adsorbed metal ions were separated and dried at room temperature for 24 h and also analyzed via UV–vis for solids.

For a standard sample (Blank), the microspheres were swollen with distilled water and deposited in the same metal ion solutions.

Anionic XO can form an aggregate through electrostatic interactions with cationic chitosan, and the XO aggregate has the potential to exhibit new sensing properties. Here we describe the development of a colorimetric chemosensor allowing naked eye detection of different metal cations in aquatic media by combining XO and C.^44^

According to Li et al.,^66^ although fluorescence detection and/or colorimetry are sensitive methods for pollutant analysis, their application is restricted to the analysis of wastewater. Therefore, we prepared a new material composed of clay mineral/chitosan interspersed with a colorimetric molecule, where the XO was inserted into the interlayer space of the clay mineral (Figure S15).

### 3.4 Ecotoxicological Biological Assays

**Figure 10:**
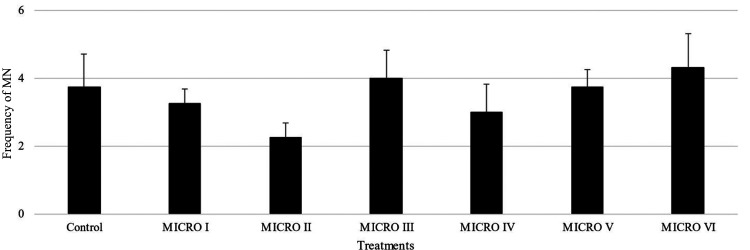
Frequencies of micronucleated (MN) erythrocytes in peripheral blood of zebrafish treated with microspheres (I, II, III, IV, V and IV). *N* = 35 (5 animals per treatment). 10,000 cells were analyzed per treatment. Values are the mean and standard deviation.

The SapCSL microspheres were tested in toxicity assays. At the end of exposure to the different microspheres, no zebrafish deaths were observed. In addition, the animals exposed to the microspheres had micronucleus frequencies that did not differ from those of the negative control group, revealing the absence of genotoxicity (Figure 10).

At the end of adsorption, concentrations of 16.7, 33.4, 50, 66.7, and 100 μg/mL were obtained, respectively, resulting in the removal of 66.7% from all solutions. The cell cultures treated with those microspheres showed significantly higher cell viability (IC_50_ = 124.3 ± 13.0 μg/mL) than those treated with chromium (IC_50_ = 89.9 ± 3.6 μg/mL), proving the potential use of the microspheres (Figure S16).

The zebrafish (*Danio rerio*) is widely recognized as a versatile and efficient experimental model due to its unique characteristics. This species possesses a fully sequenced genome and orthologous genes to humans, which facilitate studies of genotoxicity and toxicology. Additionally, it stands out for its small size, high reproductive capacity, rapid development, and ease of maintenance under laboratory conditions.^67^ These features make the zebrafish an ideal model, compatible with the 3Rs concept (replacement, reduction, and refinement), and it has been extensively studied in fields such as genetics, physiology, and environmental toxicology.^68^

The micronucleus (MN) test, widely used to evaluate the mutagenic effects of chemical agents, is one of the tools applied to zebrafish. This test detects cytogenetic damage, resulting in the formation of micronuclei containing acentric chromosomal fragments or whole chromosomes. It can be conducted using in vivo or in vitro approaches and is regulated by specific guidelines for mammals (OECD 474, 2016, and OECD 487, 2023, respectively). However, there are still no specific standards for its application in fish.

In fish studies, the MN test has been widely applied due to its simplicity, low cost, high sensitivity, and applicability under both laboratory and field conditions.^69^ These assays identify genetic damage such as chromosomal breaks, chromosomal loss, nondisjunction events, and DNA repair in various cell types or organs, such as the brain, liver, gills, testes and ovaries.^68^ Zebrafish have been extensively used in these studies to investigate the formation of micronuclei caused by traditional and emerging pollutants.

## 4 Conclusions

In conclusion, the development of chitosan and chitosan/poly(vinyl alcohol) (PVA) microspheres containing kaolinite and synthetic saponite clays in both single and double layers showed their potential use as adsorbents of metals such as Ni^2+^, Cr^3+^, and Cr^6+^ and for the removal of Ag nanoparticles from aqueous solutions. The incorporation of neutral and synthetic cationic clays played a crucial role in modifying the properties of the microspheres, including their water uptake capacity, swelling behavior and stability.

The experiments conducted with different metals revealed the versatility of the polymer/clay bionanocomposites, indicating their potential for a wide range of applications. The high adsorption efficiency and selectivity of the microspheres make them an attractive option for the removal of toxic metals from contaminated water. Furthermore, the potential use of these systems for controlled release of agrochemicals and pharmaceuticals is also a promising area for future studies.

The immobilization of XO in lamellar clay mineral matrices (natural or synthetic) can increase the selectivity of chromophores in comparison with other detection methods, making it very attractive for application in colorimetric sensors.

The ecotoxicological biological assays conducted with zebrafish (*Danio rerio*) and with HaCat human cells revealed the absence of toxicity. The experiments confirm that microspheres promoted significantly cell viability (IC_50_ = 124.3 ± 13.0 μg/mL) after treatment by adsorption strategy, confirming the efficiency of the process.

Overall, the study of chitosan and chitosan/PVA microspheres containing clays demonstrated their potential as effective and environmentally friendly adsorbent materials for removing metal ions and nanoparticles. Further studies can focus on optimizing the preparation method and exploring the use of these microspheres in other applications such as controlled release or wastewater treatment systems.

The inclusion of synthetic cationic clay improved the adsorption of cationic species by enhancing electrostatic interactions, while kaolinite contributed to more stable composites with a moderate adsorption capacity of cationic pollutants. Together, these modifications enable the development of versatile microspheres with tailored adsorption properties for advanced material applications like sensors, adsorbents, and controlled release of agrochemicals, among others.

## Supporting Information Available

Additional flowcharts of the experimental setup, characterizations, and mathematical modeling from adsorption kinetics tables with data resulted from each treatment for various samples (PDF)
