# Supplementary material for: Impact of Cationic and Neutral Clay Minerals’ Incorporation in Chitosan and Chitosan/PVA Microsphere Properties
Source: ACS Appl Mater Interfaces. 2025 Mar 28;17(14):21189–205. doi: 10.1021/acsami.4c22323 (PMC11986906; doi:10.1021/acsami.4c22323)
Supplement: Supplementary file 1 — am4c22323_si_001.pdf [file am4c22323_si_001.pdf]

# SUPPORTING INFORMATION

## Impact of cationic and neutral clay minerals' incorporation in chitosan and chitosan/PVA microsphere properties.


**Suelen Delfino Souza, Hugo Fernando Meira dos Santos, Larissa Fernandes Bonfim, Iara Silva Squarisi, Tábata Esperandim, Liziane Marçal, Denise Crispim Tavares, Emerson Henrique de Faria*.**

*Grupo de Pesquisas em Materiais Lamelares Híbridos (GPMatLam), Universidade de Franca (Unifran), Av. Dr. Armando Salles Oliveira, 201 Parque Universitário, Franca-SP, 14404-600, Brazil*

*Corresponding author: Emerson H. De Faria

e-mail: eh.defaria@gmail.com or emerson.faria@unifran.edu.br


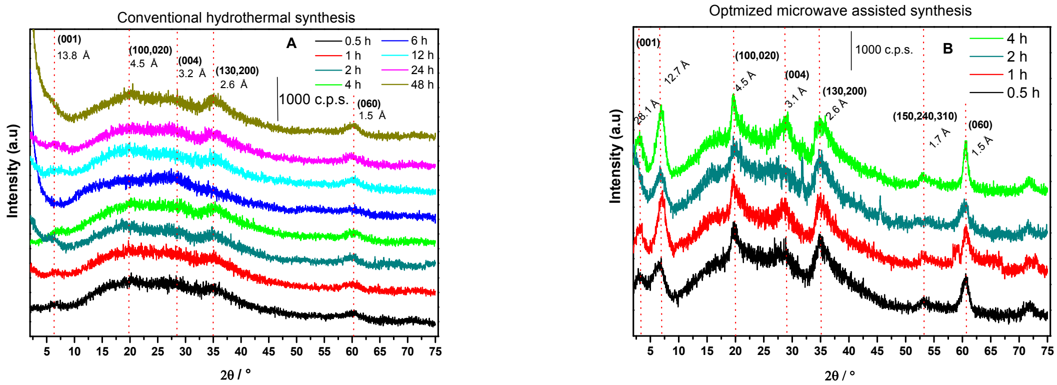


**Figure S1:** XRD of saponite hydrothermal conventional synthesis (A) and optimized microwave assisted synthesis (B)

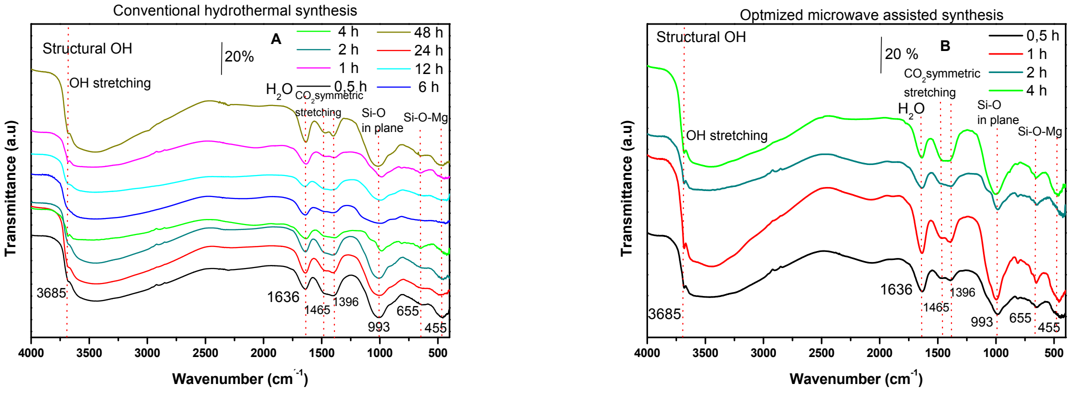


**Figure S2:** FTIR of saponite hydrothermal conventional synthesis (A) and optimized microwave assisted synthesis (B)

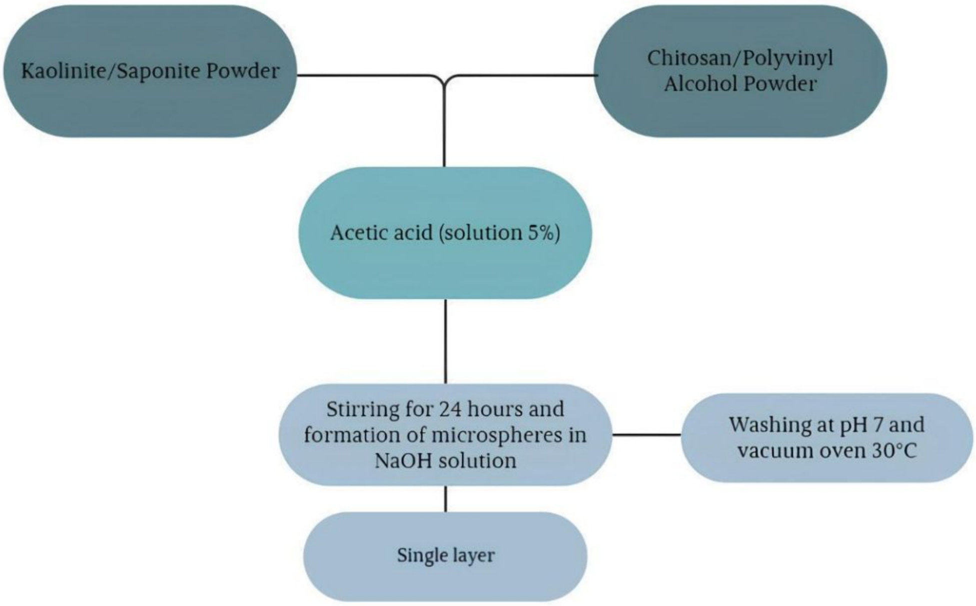


**Figure S3:** Flowchart detailing the single layer microsphere synthesis.

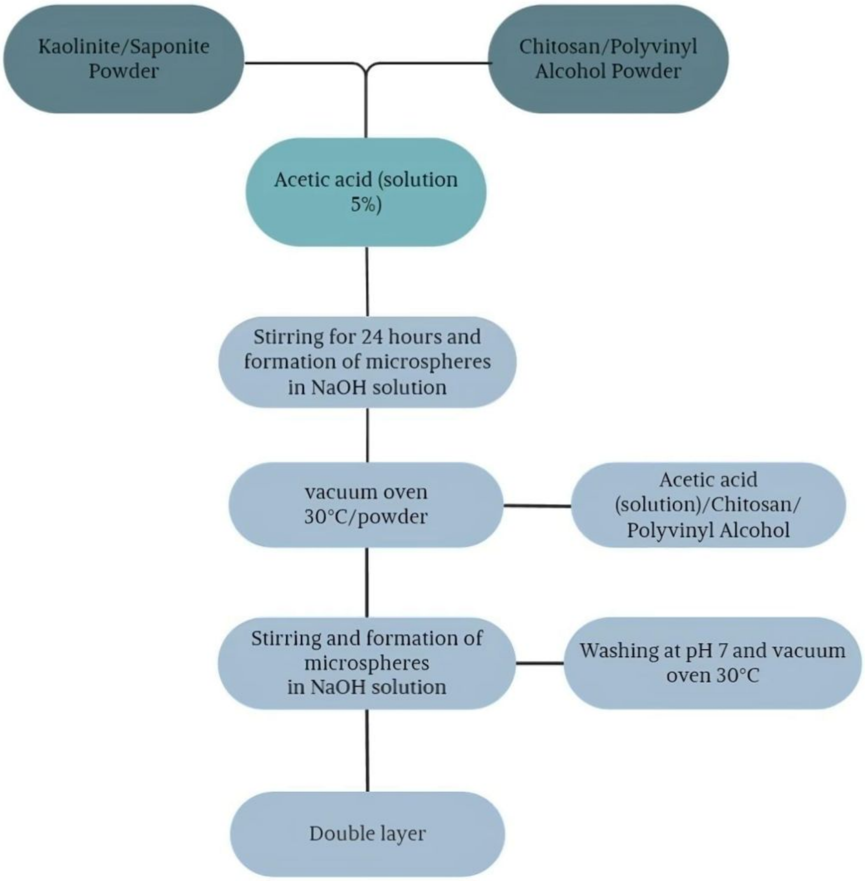


**Figure S4:** Flowchart detailing the double layer microsphere synthesis.

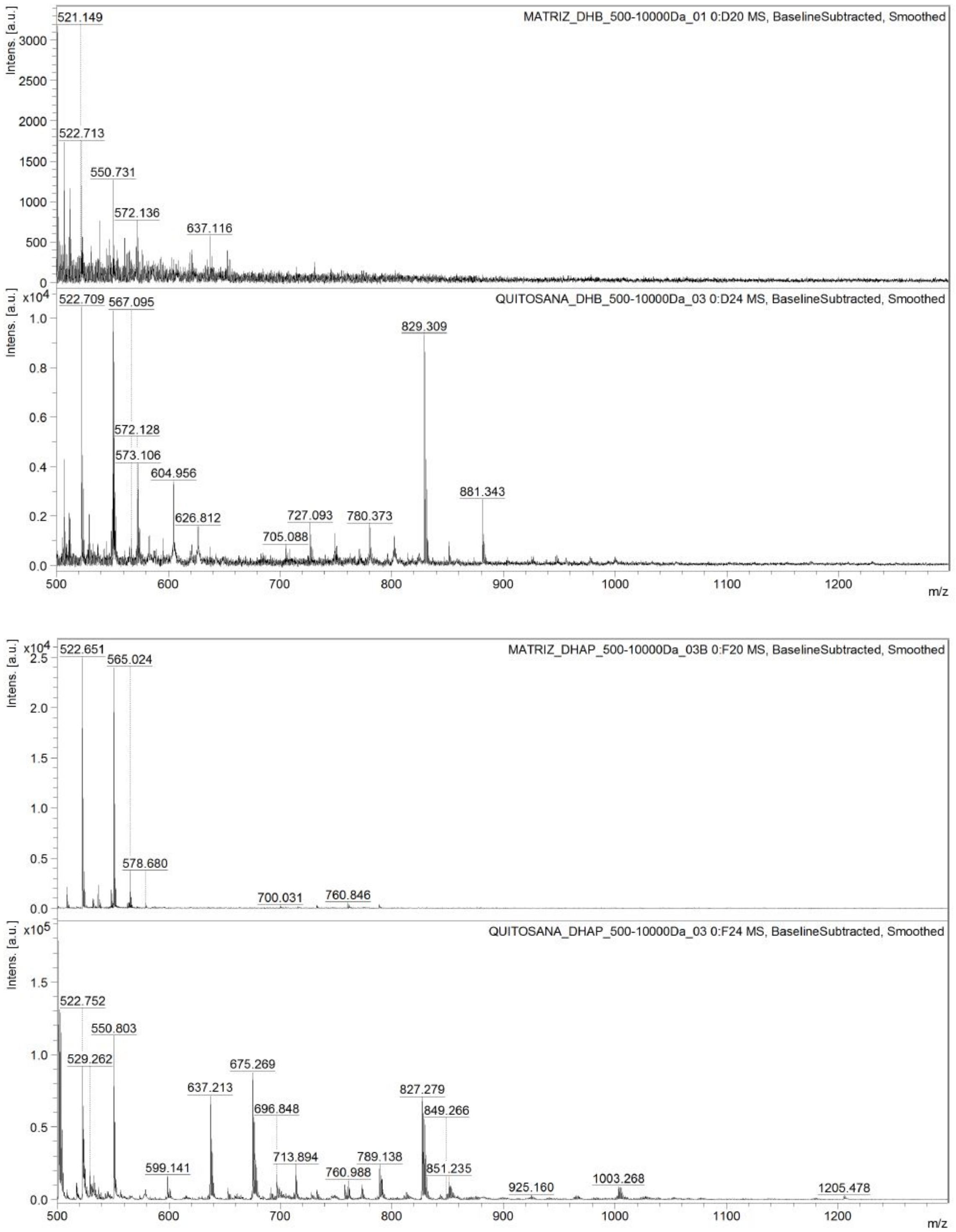


**Figure S5:** MALDI-TOF mass spectrometry analysis of commercial chitosan hydrolysate. Amongst chitosan hydrolysates, the molecular weight of CTSN-P30 derived from the hydrolysis of HMWC (1%, w/v) in acetic acid (2%) hydrolysates at 25ºC was determined by MALDI-TOF mass spectrometry analysis ranging from 500 to 10,000 m/z.

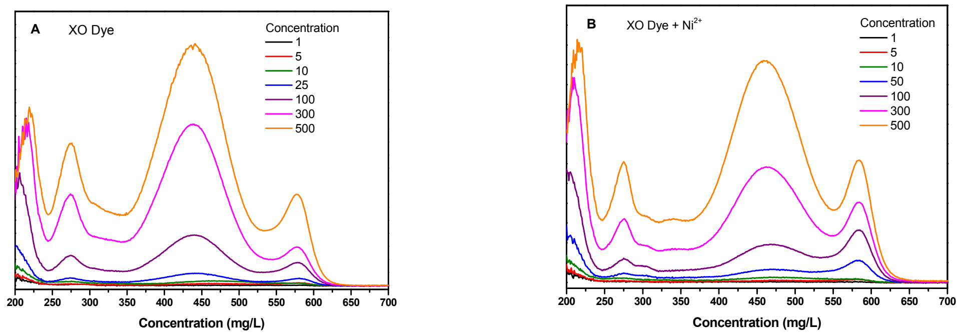


**Figure S6.** Molecular absorption spectroscopy in the ultraviolet-visible region (UV-Vis spectroscopy) of (A) concentration Orange xylenol and (B) Orange xylenol $Ni^{2+}$ ions.

**Table S1**. Kinetic parameters for MB dye.

| Microspheres | qt (mg/g) | Maximum removal (min) | Removal (%) |
|---|---|---|---|
| KaolCSL | 1.14 | 2880 | 53.30 |
| KaolCPSL | 0.96 | 2880 | 49.00 |
| KaolCDL | 0.54 | 2880 | 35.10 |
| KaolCPDL | 0.82 | 2880 | 45.10 |
| SapCSL | 1.05 | 2880 | 51.20 |
| SapCPSL | 1.04 | 2880 | 50.90 |
| SapCDL | 0.99 | 2880 | 49.70 |
| SapCPDL | 1.10 | 2880 | 52.40 |

**Table S2**. Kinetic parameters for $Ni^{2+}$.

| Microspheres | qt (mg/g) | Maximum removal (min) | Removal (%) |
|---|---|---|---|
| KaolCSL | 4.78 | 2880 | 82.70 |
| KaolCPSL | 4.18 | 1440 | 80.70 |
| KaolCDL | 1.35 | 2880 | 57.40 |
| KaolCPDL | 3.10 | 2880 | 75.60 |

| | | | |
|---|---|---|---|
| SapCSL | 3.49 | 2880 | 77.70 |
| SapCPSL | 3.13 | 2880 | 75.80 |
| SapCDL | 2.89 | 2880 | 74.30 |

**Table S3**. Kinetic parameters for $Cr^{3+}$ and $Cr^{6+}$.

| Microspheres | qt (mg/g) | Maximum removal (min) | Removal (%) |
|---|---|---|---|
| SapCSL$Cr^{3+}$ | 1006.43 | 15 | 51.20 |
| SapCPSL$Cr^{3+}$ | 1762.38 | 90 | 91.70 |
| SapCSL$Cr^{6+}$ | 17.26 | 30 | 14.10 |
| SapCPSL$Cr^{6+}$ | 19.63 | 120 | 19.68 |

**Table S4**. Kinetic parameters for AgNP

| Microspheres | qt (mg/g) | Maximum removal (min) | Removal (%) |
|---|---|---|---|
| SapCSL | 0.03 | 15 | 3.30 |
| SapCPSL | 0.90 | 10080 | 99.55 |

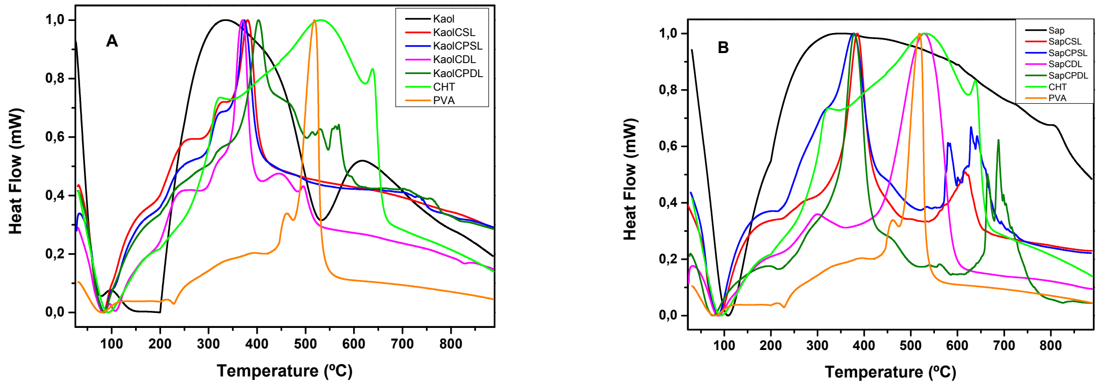


**Figure S7.** DSC curves of (A) Kaol and (B) Sap and grinded microspheres resulted from PVA and chitosan reaction.

**Table S5**: Kinetics parameters for MB dye and Nickel

| Model/parameters | Adsorbents | | | | | | | |
|---|---|---|---|---|---|---|---|---|
| | KaolCSL | | KaolCPSL | | KaolCDL | | KaolCPDL | |
| | MB | Ni | MB | Ni | MB | Ni | MB | Ni |
| pseudo first order | | | | | | | | |
| qt (mg/g) | 0.365 | 1.767 | 0.371 | 2.309 | 0.292 | 0.712 | 0.512 | 0.718 |
| K (min-1 ) | 0.000 | 0.000 | 0.000 | 0.000 | 0.000 | 0.000 | 0.000 | 0.000 |
| $R^2$ | 0.066 | 0.021 | 0.115 | 0.112 | 0.271 | 0.086 | 0.498 | 0.016 |
| $x^2$ | 0.090 | 1.674 | 0.062 | 2.168 | 0.014 | 0.244 | 0.019 | 0.486 |
| | | | | | | | | |
| pseudo second order | | | | | | | | |
| qt (mg/g) | 0.413 | 1.823 | 0.418 | 2.332 | 0.335 | 0.764 | 0.574 | 0.768 |
| K (g/mg.min) | 1.000 | 1.000 | 1.000 | 1.000 | 1.000 | 1.000 | 1.000 | 1.000 |
| $R^2$ | 0.241 | 0.048 | 0.325 | 0.153 | 0.544 | 0.177 | 0.747 | 0.068 |
| $x^2$ | 0.073 | 1.628 | 0.047 | 2.069 | 0.009 | 0.220 | 0.009 | 0.460 |
| | | | | | | | | |
| Intraparticle Diffusion | | | | | | | | |
| C | 0.089 | 0.753 | 0.135 | 1.135 | 0.168 | 0.440 | 0.361 | 0.144 |
| Kdif o (g/mg.min$^{1/2}$) | 0.021 | 0.078 | 0.017 | 0.066 | 0.008 | 0.018 | 0.010 | 0.045 |
| $R^2$ | 0.964 | 0.718 | 0.917 | 0.326 | 0.755 | 0.212 | 0.566 | 0.840 |
| $x^2$ | 0.003 | 0.480 | 0.006 | 1.646 | 0.005 | 0.211 | 0.016 | 0.079 |
| | | | | | | | | |
| Elovich | | | | | | | | |
| α (mg g-1 min-1 ) | 0.038 | 0.785 | 0.054 | 0.658 | 0.186 | 0.539 | 1.934 | 0.095 |
| β (mg.g-1 ) | 7.551 | 2.644 | 8.784 | 0.429 | 17.934 | 7.673 | 14.082 | 4.270 |
| $R^2$ | 0.863 | 0.435 | 0.910 | 0.593 | 0.911 | 0.340 | 0.982 | 0.512 |
| $x^2$ | 0.013 | 0.966 | 0.006 | 0.992 | 0.002 | 0.176 | 6.733x10 4 | 0.241 |

| Model/parameters | Adsorbents | | | | | | | |
|---|---|---|---|---|---|---|---|---|
| | SapCSL | | SapCPSL | | SapCDL | | SapCPDL | |
| | MB | Ni | MB | Ni | MB | Ni | MB | Ni |
| pseudo first order | | | | | | | | |
| qt (mg/g) | 0.421 | 1.194 | 0.293 | 1.055 | 0.403 | 1.855 | 0.380 | 1.747 |
| K (min-1 ) | 0.000 | 0.000 | 0.000 | 0.000 | 0.000 | 0.000 | 0.000 | 0.000 |
| $R^2$ | 0.089 | 0.046 | 0.010 | 0.009 | 0.122 | 0.004 | 0.074 | 0.007 |
| $x^2$ | 0.102 | 0.824 | 0.111 | 0.991 | 0.075 | 0.544 | 0.094 | 4.977 |
| | | | | | | | | |
| pseudo second order | | | | | | | | |
| qt (mg/g) | 0.471 | 1.244 | 0.341 | 1.107 | 0.453 | 0.647 | 0.428 | 1.776 |
| K (g/mg.min) | 1.000 | 1.000 | 1.000 | 0.052 | 1.000 | 1.000 | 1.000 | 1.000 |
| $R^2$ | 0.295 | 0.077 | 0.164 | 0.051 | 0.355 | 0.647 | 0.244 | 0.017 |
| $x^2$ | 0.079 | 0.797 | 0.094 | 0.949 | 0.055 | 0.032 | 0.076 | 4.853 |
| | | | | | | | | |
| Intraparticle Diffusion | | | | | | | | |
| C | 0.137 | 0.553 | 0.007 | 0.281 | 0.149 | 0.004 | 0.021 | $9.989x10^{24}$ |
| Kdif o (g/mg.$min^{1/2}$) | 0.021 | 0.048 | 0.022 | 0.060 | 0.189 | 0.421 | 0.865 | 0.147 |
| $R^2$ | 0.832 | 0.517 | 0.940 | 0.743 | 0.850 | 0.736 | 0.941 | 0.963 |
| $x^2$ | .0.018 | 0.416 | 0.006 | 0.257 | 0.012 | 0.004 | 0.006 | 0.182 |
| | | | | | | | | |
| Elovich | | | | | | | | |
| α (mg g-1 min-1 ) | 0.008 | 0.752 | 0.021 | 0.139 | 0.052 | 0.098 | 0.041 | 0.120 |
| β (mg.g-1 ) | 0.453 | 1.372 | 7.016 | 2.873 | 7.660 | 5.457 | 7.349 | 1.097 |
| $R^2$ | 0.942 | 0.378 | 0.819 | 0.564 | 0.967 | 0.801 | 0.865 | 0.754 |
| $x^2$ | 0.006 | 0.537 | 0.020 | 0.435 | 0.002 | 0.965 | 0.014 | 1.212 |

**Table S6**: Kinetic parameters for *$Cr^{3+}$*, *$Cr^{6+}$and AgNP*

| Model/parameters | Adsorbents | | | | | |
|---|---|---|---|---|---|---|
| | SapCSL | | SapCPSL | | SapCSL | SapCPSL |
| | $Cr^{3+}$ | $Cr^{6+}$ | $Cr^{3+}$ | $Cr^{6+}$ | AgNP | AgNP |
| pseudo first order | | | | | | |
| qt (mg/g) | 725.347 | 8.528 | 1431.598 | 11.380 | 0.015 | 0.684 |
| K (min-1 ) | 204.810 | 60.266 | 1120.236 | 2.586 | 86.268 | 0.006 |
| $R^2$ | 0.5116 | 0.109 | 0.8108 | 0.342 | 0.092 | 0.709 |
| $x^2$ | 38517.998 | 28.393 | 39024.331 | 17.542 | $7.559x10^{-5}$ | 0.020 |
| | | | | | | |
| pseudo second order | | | | | | |
| qt (mg/g) | 725.356 | 8.528 | 1444.580 | 11.730 | 0.01458 | 0.756 |
| K (g/mg.min) | $7.023x10^{44}$ | 0.436 | 0.012 | 0.320 | $4.467x10^{21}$ | 0.009 |
| $R^2$ | 0.511 | 0.112 | 0.813 | 0.356 | 0.089 | 0.786 |
| $x^2$ | 38517.998 | 28.281 | 38460.616 | 17.146 | $7.582x10^{-5}$ | 0.015 |
| | | | | | | |
| Intraparticle Diffusion | | | | | | |
| C | 618.982 | 7.106 | 1275.776 | 10.659 | 0.017 | 0.157 |
| Kdif o (g/mg.min$^{1/2}$) | 4.553 | 0.087 | 3.621 | 0.000 | 0.000 | 0.008 |
| $R^2$ | -0.054 | -0.076 | -0.088 | -0.104 | -0.202 | 0.855 |
| $x^2$ | 83110.644 | 224592.771 | 17.667 | 34.308 | $1.002x10^{-4}$ | 0.010 |
| | | | | | | |
| Elovich | | | | | | |
| α (mg g-1 min-1 ) | $3.018 x10^{11}$ | $8.369x10^{44}$ | $1.024x10^{45}$ | $3.864x10^{44}$ | 0.000 | 0.049 |
| β (mg.g-1 ) | 0.036 | 3.494 | 0.073 | 9.587 | 68.721 | 11.224 |
| $R^2$ | 0.563 | 0.120 | 0.799 | 0.337 | -2.784 | 0.811 |
| $x^2$ | 34447.622 | 28.029 | 41308.672 | 17.667 | $3.152x10^{-4}$ | 0.014 |

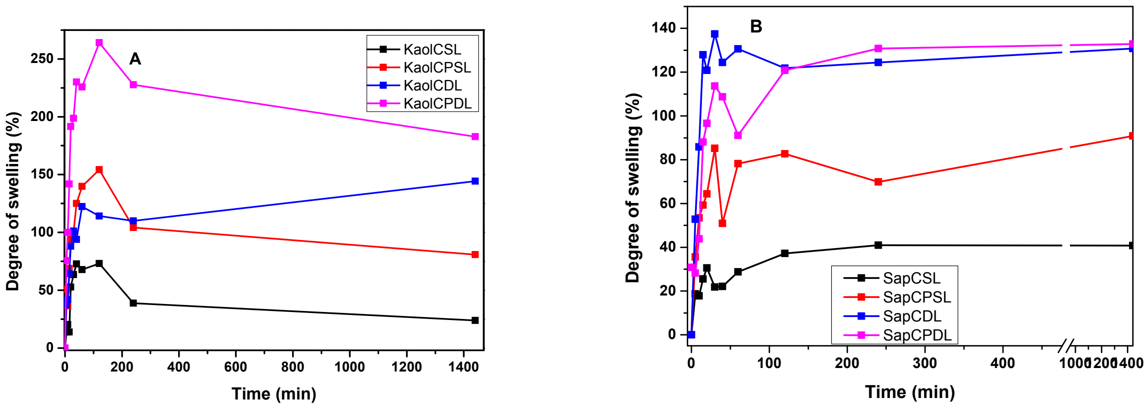


**Figure S8.** Water uptake by (A) Kaol microspheres and (B) Sap microspheres.

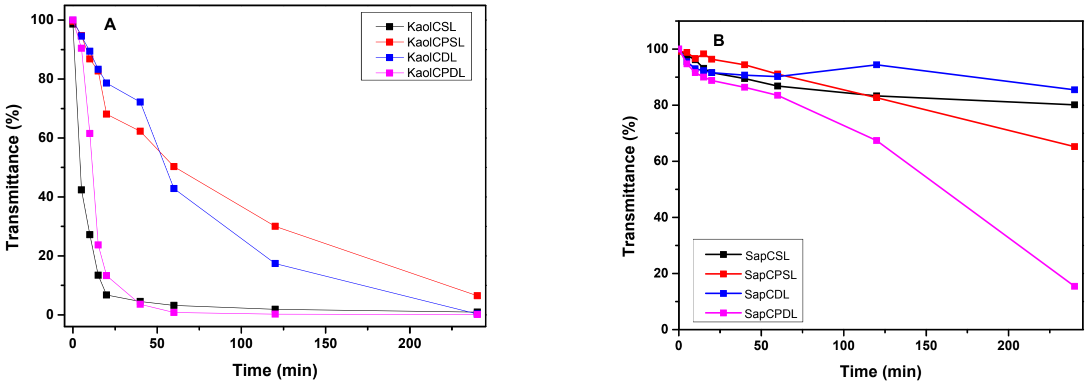


**Figure S9.** Kinetic study results from acid stability testing of microspheres containing (A) Kaol and (B) Sap.

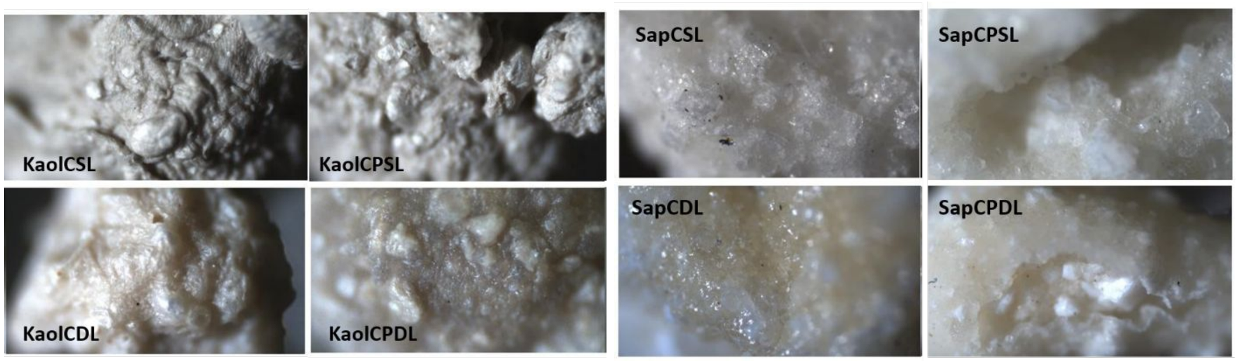


**Figure S10.** Images of microspheres.

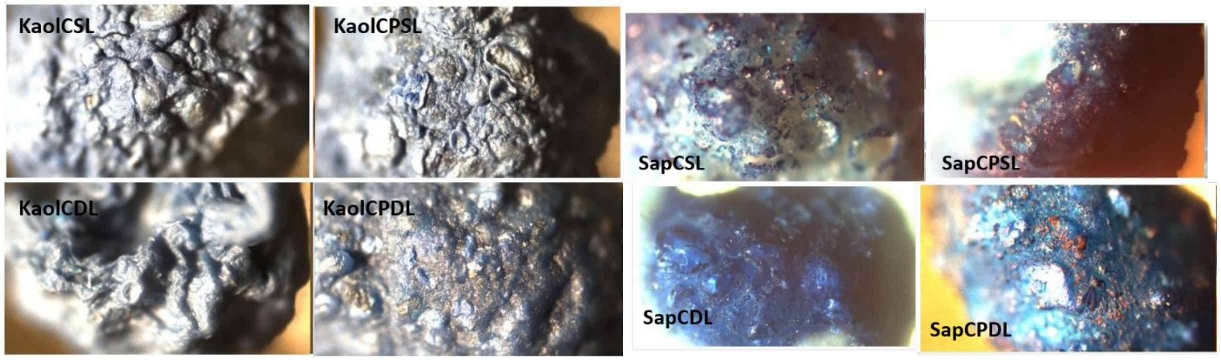


**Figure S11.** Images taken after the MB adsorption.

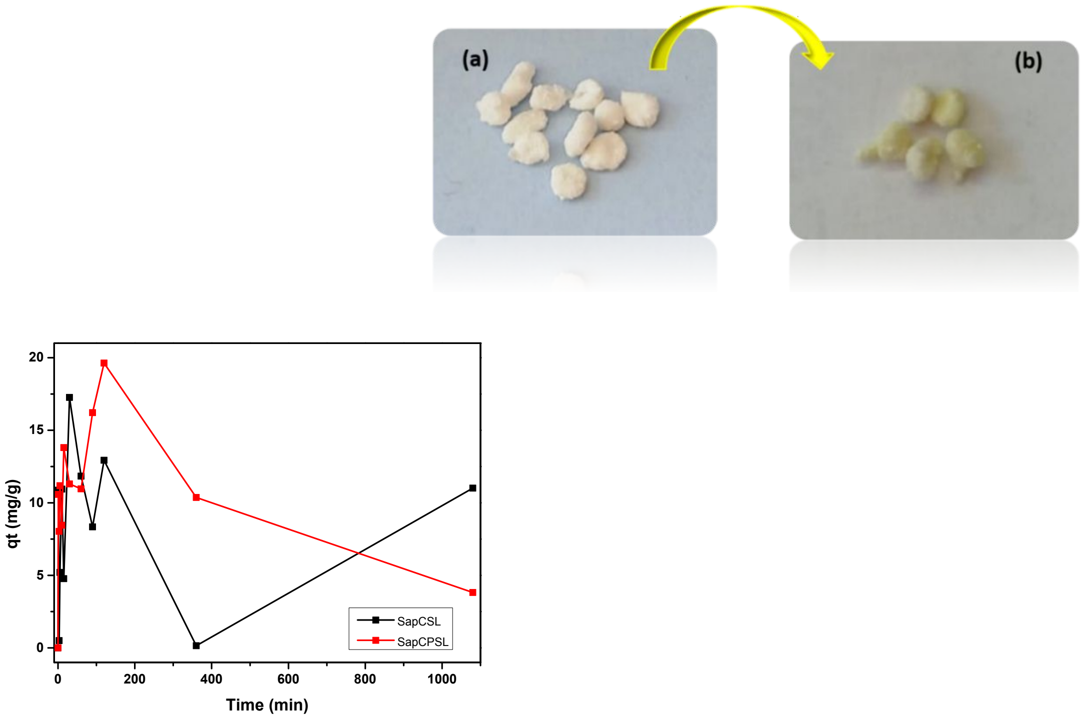


**Figure S12.** Adsorption Kinetics (condition for $Cr^{6+}$: 60.0 mg $L^{-1}$ , $t$ = 0–2880 min, used as adsorbate, and 100.0 mg of each microsphere as adsorbent), and image of the bionanocomposites: (a) SapCSL or SapQCPSL before the $Cr^{6+}$ adsorption process; and (b) after the.process.

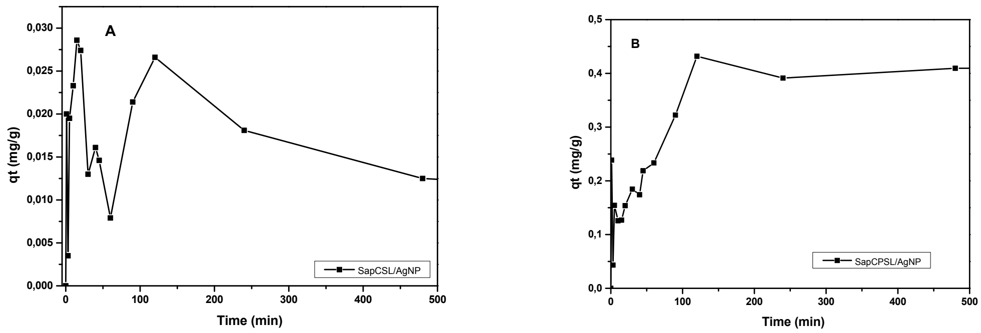


**Figure S13.** Adsorption Kinetics (condition for AgNP) $C_0$ = 1.68 mg $L^{-1}$ $t$ = 0–1000 min solution as adsorbate and 100.0 mg of-microsphere as adsorbent).

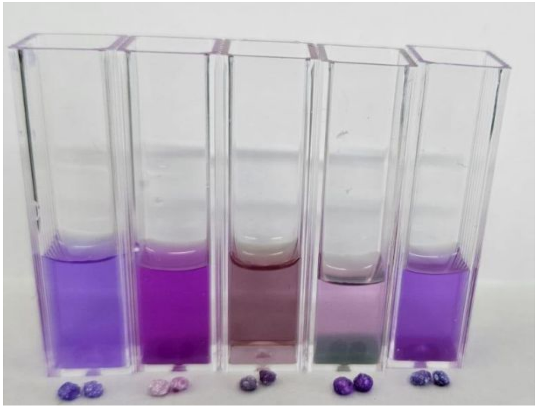

**Figure S14.** Image demonstrating the change in color of the microspheres after adsorption.

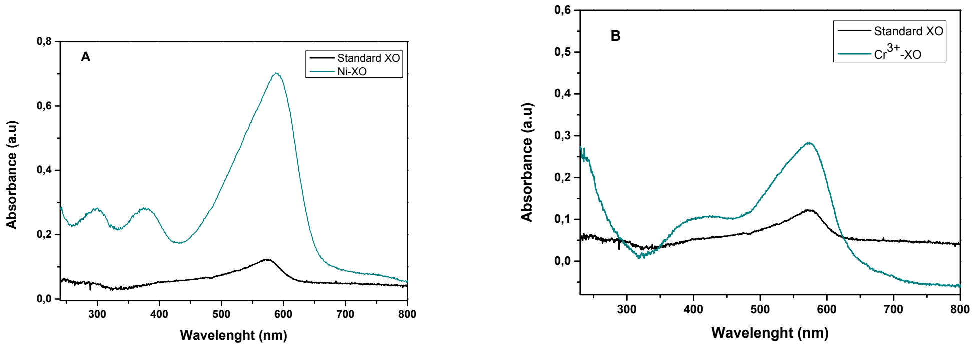

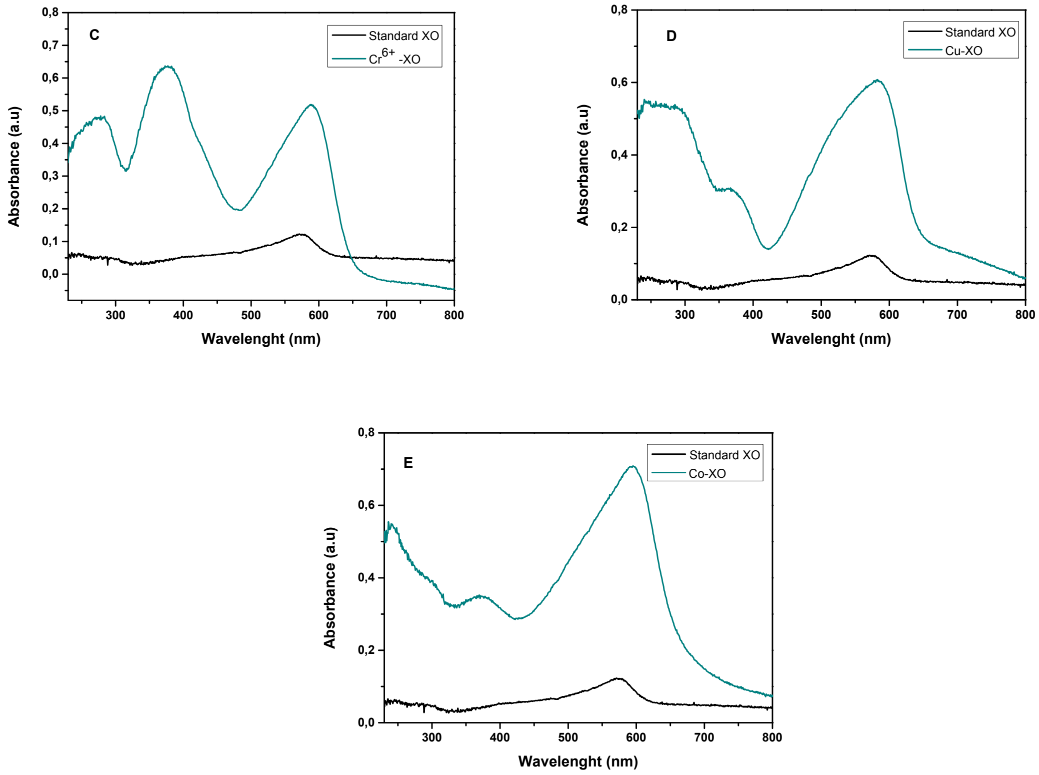


**Figure S15.** Molecular absorption spectroscopy in the ultraviolet-visible region (UV-vis spectroscopy of (A) $Ni^{2+}$, (B) $Cr^{3+}$, (C) $Cr^{6+}$ (D) $Cu^{2+}$ and (E) $Co^{2+}$.

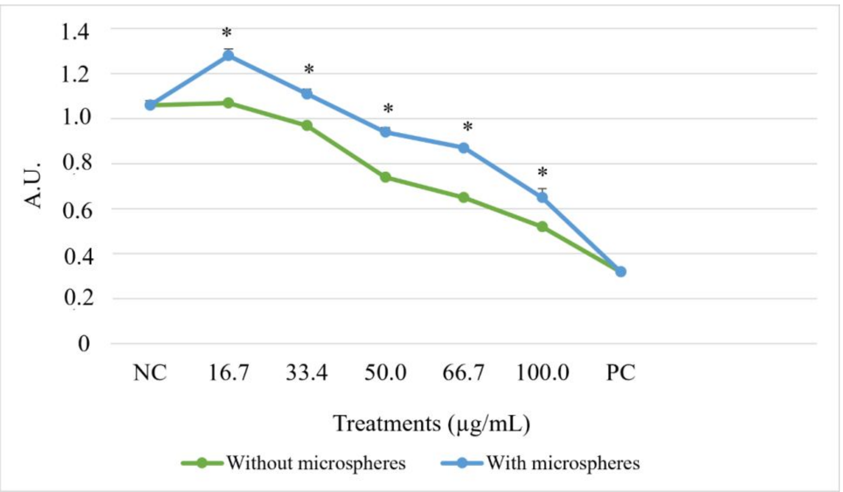


**Figure S16.** Cell viability absorbance observed in HaCat cells after exposure to chromium solutions with and without pretreatment with microspheres.

A.U.: absorbance units; NC: negative control; PC: positive control (dimethyl sulfoxide, 25%). $IC_{50}$=124.3 ± 13.0 µg/mL-pretreatment with microspheres. $IC_{50}$=89.9 ± 3.6 µg/mL-without pretreatment with microspheres. Values are mean ± SD. *Significantly different from groups without microspheres ($p$<0.05).
